# Myocyte Enhancer Factor 2c Regulates Dendritic Complexity and Connectivity of Cerebellar Purkinje Cells

**DOI:** 10.1007/s12035-018-1363-7

**Published:** 2018-10-01

**Authors:** Sandhya Prakash Kamath, Albert I. Chen

**Affiliations:** 1 School of Biological Sciences, Nanyang Technological University (NTU), Singapore, 637551 Singapore; 2 A*STAR, Institute of Molecular and Cell Biology, Singapore, 138673 Singapore; 3 School of Life Sciences, University of Warwick, Coventry, CV4 7AL UK

**Keywords:** Mef2c, Cerebellum, Purkinje cells, GABAergic neurons, Dendrites, Spines

## Abstract

Mef2c haploinsufficiency is implicated in behavioral deficits related to autism, schizophrenia, and intellectual disability. Although perturbations in the cerebellum, notably Purkinje cells, have been linked to these neurological disorders, the underlying mechanisms remain poorly understood. In this study, we investigated the roles of Mef2c in cerebellar Purkinje cells during the first three weeks of postnatal development. Our analysis revealed that in comparison to other members of the Mef2 family, Mef2c expression is limited to postnatal Purkinje cells. Because the role of Mef2c has not been assessed in GABAergic neurons, we set out to determine the functional significance of Mef2c by knocking down the expression of Mef2c selectively in Purkinje cells. We found that the loss of Mef2c expression during the first and second postnatal week results in an increase in dendritic arborization without impact on the general growth and migration of Purkinje cells. The influence of Mef2c on dendritic arborization persists throughout the first three weeks, but is most prominent during the first postnatal week suggesting a critical period of Mef2c activity. Additionally, the loss of Mef2c expression results in an increase in the number of spines accompanied by an increase in Gad67 and vGluT1 puncta and decrease in vGluT2 puncta. Thus, our results reveal the specific expression and functional relevance of Mef2c in developing Purkinje cells and offer insight to how disruption of the expression of Mef2c in a GABAergic neuronal subtype may lead to pathogenesis of cerebellar-associated disorders.

**Electronic supplementary material:**

The online version of this article (10.1007/s12035-018-1363-7) contains supplementary material, which is available to authorized users.

## Introduction

The transcription factor myocyte enhancer factor 2c (Mef2c) has been identified in human genetic analysis as a susceptibility gene for a number of neurological disorders. The partial loss of Mef2c, referred to as Mef2c haploinsufficiency syndrome, has been linked to autism spectrum disorder (ASD), schizophrenia, and intellectual disability, which are thought to be caused by impairments during early stages of neural development [1–3]. In addition to regulation of the development of muscle, bone, and lymphocytes [4], Mef2c orchestrates neuronal differentiation and survival as well as synapse formation in rodents [5–12]. Mef2c is expressed in many parts of the human and rodent brain [13–15], and activation of the transcriptional activity of Mef2 family members by neurotrophin and calcium influx controls a number of excitatory synapses [6, 16]. Despite the emerging role of Mef2c as an important activity-dependent regulator of neural processes, details of its expression pattern and function have only been assessed in select regions of the brain. Mouse genetic studies have identified key roles of Mef2 in neurons of the cerebral cortex and hippocampus [5, 9–12], but consequences of deletion of Mef2 genes on the development of the cerebellum have not yet been examined or reported. Moreover, the role of Mef2c in GABAergic neurons is unknown.

There is growing support for the link between dysfunction of the cerebellum and neurological disorders such as ASD [17–19], and both human and animal studies point to a reduction in the number or disrupted function of Purkinje cells [20–23]. Purkinje cells are GABAergic projection neurons located in the cerebellar cortex that receive inhibitory input from molecular layer interneurons and excitatory input from parallel and climbing fibers [24, 25]. Generation of Purkinje cells in mice begins at embryonic day 10.5 when they begin to proliferate, migrate, and differentiate until birth [26, 27]. Dendritic formation and spinogenesis, on the other hand, involve the addition and retraction of processes that occur during the first 3 weeks of postnatal development [28]. Purkinje cell dendrites develop from multiple perisomatic processes to a single apical stem during the first postnatal week, and the rapid branching of the apical stem dendritic tree takes place during the second week [28]. Disruptions in one or more of these events during development could perturb the function of Purkinje cells leading to an imbalance of excitation and inhibition, which is often associated with neurological disorders [29, 30]. Thus, the identification and characterization of factors that coordinate complex processes such as dendritic outgrowth and synaptic organization in Purkinje cells may provide insight into how perturbations in cerebellar circuits could lead to disorders associated with the cerebellum.

In this study, we characterize the spatio-temporal expression pattern of Mef2c in the mouse cerebellum and show that Mef2c is specifically expressed in Purkinje cells in the postnatal cerebellar cortex. Since the onset of Mef2c expression coincides with a period of rapid structural changes in Purkinje cell dendrites, we explored its role in dendritic morphogenesis and synapse formation. Using a Purkinje cell-specific promoter to drive shRNA targeted against Mef2c and selectively label individual Purkinje cells, we analyzed consequences of Mef2c knockdown during early postnatal development. We found that the loss of Mef2c expression results in an increase in dendritic complexity, and the influence of Mef2c persists throughout the first 3 weeks. Our results indicate that the first postnatal week may be a critical period of Mef2c activity because the loss of Mef2c expression during this period results in a more pronounced change in properties of Purkinje cell dendrites. We also observe that reducing the expression of Mef2c results in a modest increase in the number of distal spines, but not the length of spines. The loss of Mef2c also leads to changes in localization of vGluT1 and vGluT2 puncta on Purkinje cell dendrites, indicating disrupted input from climbing and parallel fibers. Additionally, loss of Mef2c results in an increase in Gad67 puncta on Purkinje cell dendrites and soma. Taken together, we show that the expression of Mef2c in Purkinje cells is a determinant of dendritic complexity and synaptic input and provide evidence that the perturbation of a key transcription factor linked to neurological disorders in GABAergic neurons may underlie pathogenesis of cerebellar-associated disorders.

## Materials and Methods

### Animals and Ethics Statement

All experiments conducted were approved by the Institutional Animal Care and Use Committee in accordance with the guidelines stated by the National Advisory Committee for Laboratory Animal Research. C57BL/6N mice were housed in the Biological Research Center under the management of the Agency for Science, Technology and Research and Animal Research Facility, LKC School of Medicine, Nanyang Technological University, Singapore. Mice were provided ad libitum access to food and water and were reared on a 12-h light-dark cycle.

### Histology

For in situ hybridization, mice were euthanized using a CO_2_ chamber, the brains were extracted and incubated overnight in 4% paraformaldehyde (Sigma). On the subsequent day, fixed brains were transferred into 30% sucrose solution (1st base) and dehydrated overnight, following which they were mounted in optimal cutting temperature (Sakura Finetek) compound and frozen using dry ice. For immunohistochemistry, mice were anesthetized by intraperitoneal injection of 2.5% Avertin [0.025 g/mL of 2,2,2-tribromoethanol (Sigma), mixed with 2-methyl-2-butanol, (Sigma)]. Once they were completely sedated, cardiac perfusion was carried out first with 0.9% saline, followed by 4% paraformaldehyde. The brains were then transferred to 30% sucrose and allowed to dehydrate overnight. Once they were sunken in the sucrose bed, they were either flash frozen in optimal cutting temperature compound or used directly for vibratome sectioning.

Brains used for in situ hybridization analysis and characterization of Mef2c expression were sectioned on a cryostat in 20-μm thickness. Sections were collected on Superfrost plus microscopic slides (Thermo Fisher) and were allowed to dry in room temperature for 20 min. Sections were either used immediately or stored in − 80 °C. Tissues used for the morphological analysis of Purkinje cells were 100 μm thick and sectioned using a vibratome (Leica VT 1000 S), which were then used directly for immunohistochemical analysis or stored in tissue collection solution (TCS) [25% glycerin, 30% ethylene glycol, 1.38 g/L monosodium phosphate, and 5.48 g/L disodium phosphate (Sigma)] at − 20 °C until further use.

### In Situ Hybridization

RNA probes with approximate lengths of 900 bps were generated for all four members of the Mef2 family using the following sequences: Mef2a: 5′-CAGCCAGCTCAACATTAGCA-3′, Mef2b: 5′-GTGCTTTGTGACTGCGACAT-3′, Mef2c: 5′-TGATCAGCAGGCAAAGATTG-3′, Mef2d: 5′-CACTCCTTCCCTGGTGACAT-3′. The T7 polymerase sequence 5′-GCGCTAATACGACTCACTATAGGG-3′ was added to the 5′ end of the reverse primer. Total cerebellar RNA of mouse origin (Clontech) was reversed transcribed (Thermo Scientific) to generate template cDNA. Polymerase chain reaction (i-DNA Technology) was carried out with the primers and the resulting product was labeled with 11-digoxygenin-UTP (Roche). Chromogenic in situ hybridization was then carried out as previously described [31].

### Lentivirus Production and Intracranial Injection into Neonatal Mice

Viral particles with titers > 10^8^ TU/mL were commercially produced by Vector Builder (Cyagen Biosciences) according to the construct designs provided. The L7 plasmid was a gift from Dr. Hirokazu Hirai, Gunma University, Japan [32]. The nucleotide sequences for shRNAs used are as follows: scrambled: 5′-CCCTAAGGTTAAGTCGCCCTCG-3′ and Mef2c: 5′-GGAACAACTTCCTGGAGAAGC-3′.

Intracranial injection of lentiviruses was carried out on 1-, 3-, and 7-day-old pups as described in [33] with some differences. The pups were separated from their mothers and anesthetized on ice until immobilized. This process is done one pup at a time to avoid the basal body temperatures from dropping drastically. L7-shRNA-IRES-GFP lentivirus is first diluted to 10^6^ by mixing with 2.5 mg/mL Fast Green FCF dye (Sigma), which was used to visualize the site and spread of the virus injected. An anesthetized pup was then placed on an ice pack and its head was wiped down with a cotton swab soaked in 70% ethanol. One microliter of virus was introduced into each injection site along the late maturing lobules of the cerebellum. Bilateral injections were carried out on lobules IV–VIII with one injection on each lobule. As the skin of young mice pups was translucent, the lambda suture point was visible and served as a landmark for the injection along the medio-lateral and rostro-caudal axis of the cerebellum. The needle was inserted perpendicular to the skull at a depth of 500 μm which was then held in place for 3 min to prevent backflow. Since injections were carried out in the same litter, sterile tattoo ink was injected into the palms to distinguish the control from the experimental pups. Subsequently, all the injected pups were placed on a heating pad until they recover and were returned to their home cage where they were held until a specific experimental time point.

### Immunohistochemistry

Primary antibodies and dilutions used in this study: rabbit anti-Mef2c (1:500, Cell Signaling), mouse anti-mGluR2 (1:1500, Advanced Targeting Systems), mouse anti-NeuN (1:1000, Millipore), mouse anti-Calbindin (1:5000, Swant), goat anti-parvalbumin (1:2500, Swant), rat anti-GFP (1:1000, Nacalai Tesque), guinea pig anti-vGluT2 (1:2000, Millipore), rabbit anti-GFAP (1:500, Sigma Aldrich), and guinea pig anti-zebrin (1:1000, Frontier Institute). All secondary antibodies used were obtained from molecular probes and diluted 1:1000 prior to use.

#### For 20-μm Sections

Tissues were first permeabilized for 10 min with 0.2% Triton-X (OmniPur) diluted in phosphate buffered saline (PBS) (first base). The slides were then blocked with 3% horse serum (Invitrogen) in 0.1% Triton-X (OmniPur) in PBS. Primary antibodies were diluted in blocking buffer and added onto the slides before incubating them overnight at 4 °C. The following day, corresponding secondary antibodies were diluted 1:1000 each and incubated for 2 h at room temperature. Freshly prepared 0.1 μg/mL DAPI (Sigma) was prepared and the slides were incubated for 10 min and mounted with Prolong Gold (molecular probes). The mounting agent was allowed to set overnight away from light before subsequent confocal imaging.

#### For 100-μm Sections

Free-floating immunohistochemistry was carried out for 100-μm sections. Brain tissues sectioned using the vibratome were either stored in TCS solution as mentioned previously or immediately processed. The tissue sections were first washed with PBS (First Base) and subsequently permeabilized using 0.3% Triton-X for 1 h at room temperature. The tissue sections were then incubated for another hour in blocking solution composed of 3% horse serum and 0.1% Triton-X. Sections were stained for two nights in specific concentrations of the respective antibodies. Subsequently, tissues were stained with secondary antibodies Alexa fluor 488/555/647 and 0.1 μg/mL DAPI and mounted using Prolong Gold.

### Image Analysis and Quantification

All images in this report were captured using × 10, × 20, or × 63 oil objective on the Zeiss LSM-710 Confocal Microscope System (Axio Imager Z2). Images obtained were in an 8-bit format and imaging parameters such as laser power, digital gain, and offset were kept unchanged for each experiment. Processing was carried out using ImageJ and Fiji.

#### Intensity Measurement

In order to quantify the expression level of Mef2c in GFP^+^ Purkinje cells, z-stack images of 10 μm were acquired using a × 20 objective. Images were acquired in steps of 1 μm and compressed before analysis. The threshold for each of these channels was then independently determined as mentioned previously [34], but with slight modifications. The average pixel intensity of at least five non-overlapping regions was first calculated and the standard deviation generated was added to this value. Using the subtract function in ImageJ, background was eliminated from the image. Similar to the method used for cell counting, the elliptical tool was selected and each individual Purkinje cell was drawn and the intensity was determined for the respective channels. These intensities were then further normalized to account for the disparity in antibody staining from one experiment to the next. Average intensity values were obtained for both the control and experimental Purkinje cells from at least five images taken from two to three different slides. The number obtained from dividing average intensity of the experimental group versus the control group was used to normalize all the images.

#### vGluT1/2 and Gad67 Puncta Quantification

To analyze vGluT2 puncta on PC dendrites, z-stack images of 10-μm thickness, in steps of 0.5, were obtained using a × 63 oil objective. ImageJ was used to convert these images into maximum intensity stacks, after which threshold adjustment was carried out as mentioned above to obtain the final images. The GFP channel was converted to green and vGluT2 was converted to red. Using the RG2B plugin on ImageJ, which supports only dual-channel colocalization, yellow puncta were identified within manually drawn regions of interest, either covering the full area of the soma or the dendrites. Analyze Particle property on ImageJ was then applied to determine the ratio of the area of vGluT2 puncta in the specified dendritic region. Normalization was carried out in the same way mentioned above, but with the average number of particles in the control and experimental groups, instead of pixel intensity. All further statistical tests were carried out using GraphPad Prism.

#### Dendrite Tracing and Analysis

For morphological analysis of Purkinje cells at P14 and P21, × 20 stack images in 50- and 80-μm (steps of 1) thickness were obtained, respectively. Following the aforementioned method of threshold adjustment, each dendritic tree of Purkinje cells was traced out manually using Simple Neurite Tracer plugin on Fiji [35]. 3D-Sholl analysis using concentric circles in steps of 10 μm was conducted using the plugin on ImageJ to obtain data for maximum intersections, radii of maximum intersections, and intersection number vs distance from soma, all analyzed on GraphPad.

#### Spine Analysis

A × 63 oil immersion objective was used to take 20-μm stack images. Maximum intensity projections of this stack, taken in steps of 0.5, were generated and threshold values were calculated as previously described [34]. Images were then converted to grayscale and spines were manually counted from at least 20 independent tertiary/terminal dendrites using the Cell Counter function on ImageJ. The total number of spines was determined in 10-μm stretches of dendrite per neuron and density was calculated as the number of spines divided by 10. Lengths of individual spines were measured as the distance from the mid-line of each spine to the tip of the spine head. GraphPad was employed for subsequent *t* test analysis.

### Statistical Analysis

All data obtained was analyzed using GraphPad Prism. The pattern of data distribution and outliers was first identified for each data set using the respective tools on GraphPad. Numbers including animals used, puncta, dendrites, spines, and cells counted are stated in each figure legend. For this study, only apical Purkinje cells from lobules III–VIII of the vermis region were used for analysis. Unpaired *t* tests were used for all the experiments.

## Results

### The Expression of Mef2c RNA and Protein Is Restricted to Purkinje Cells in the Postnatal Cerebellar Cortex

**Fig. 1:**
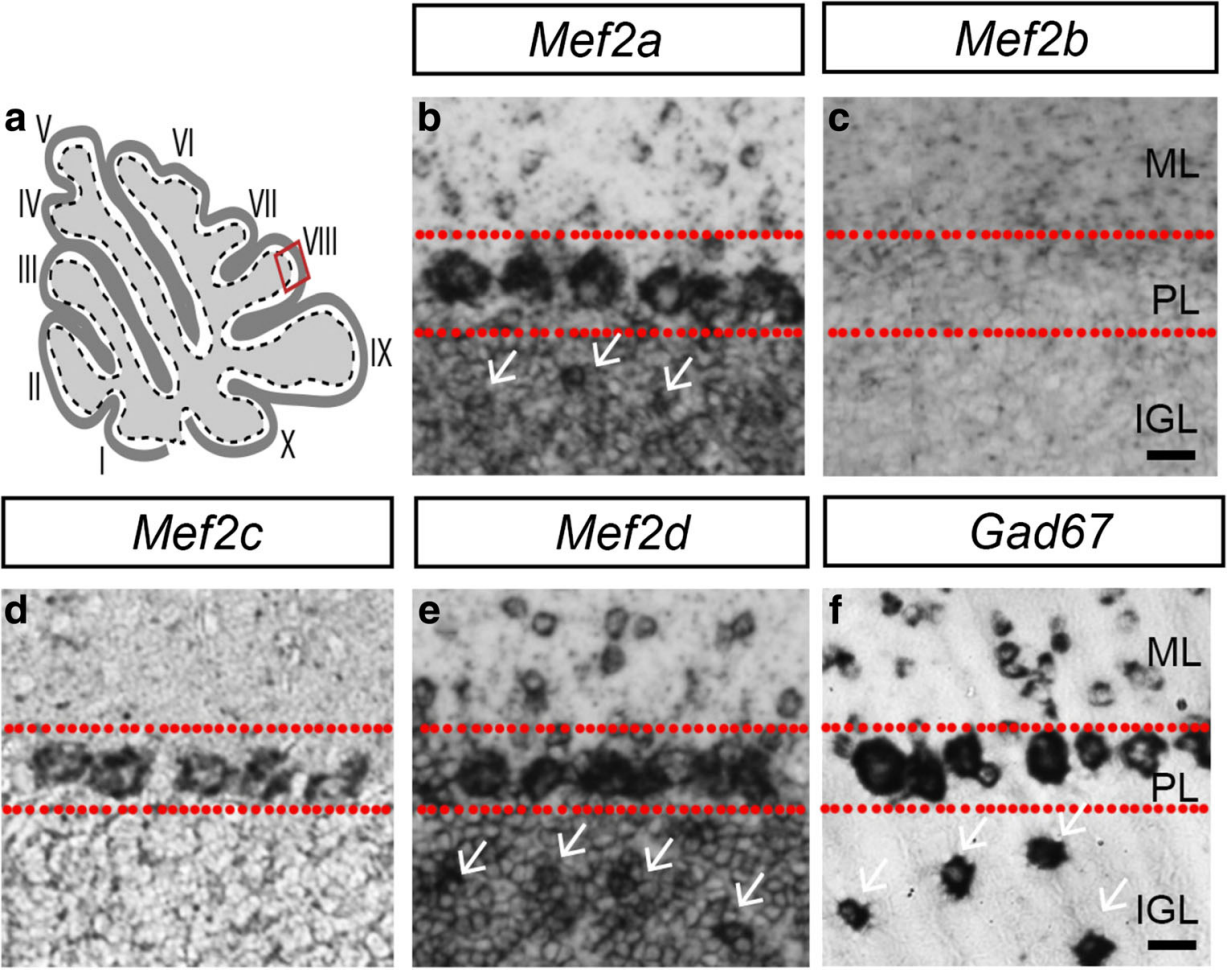
Distinct expression of *Mef2* genes in the cerebellar cortex. **a** Schematic diagram of a sagittal section of the cerebellum with red box indicating lobule VIII where subsequent images are derived from. **b** *Mef2a* expression is observed in the ML, PL and IGL layers of the cerebellar cortex (white arrows indicate presumptive Golgi cells). **c** *Mef2b* expression is not detected in any of the cerebellar cortical layers. **d** *Mef2c* expression is limited to the PL indicating specific expression in Purkinje cells. **e** *Mef2d* expression is found in the ML, PL and IGL layers (white arrows indicate presumptive Golgi cells). **f** *Gad67*, a marker for GABAergic neurons, is observed in stellate and basket cells in the ML, Purkinje cells in the PL and Golgi cells in the IGL. PL, Purkinje cell layer; ML, molecular layer; IGL, internal granule layer. Age = P60. Scale bar = 20 μm

Analysis of the expression of *Mef2* family of transcription factors in the human and mouse brain has revealed that all four *Mef2* genes are expressed in the cerebellar cortex [36], but details of the temporal and spatial expression pattern within specific neuronal subtypes are not clear. To characterize the expression of *Mef2* genes in the cerebellar cortex, we generated RNA probes specific for each homolog and performed chromogenic in situ hybridization analysis in lobule VIII of cerebellar sections obtained from postnatal day (P) 60 mice. The expression of *Mef2a* and *Mef2d* is found in the molecular layer and Purkinje cell layer, indicating that these two Mef2 family members are expressed by stellate/basket cells and Purkinje cells (Fig. 1 b, e). The widespread expression throughout the internal granular layer indicates that granule cells express both *Mef2a* and *Mef2d*, and the sparsely distributed intense expression suggests that they may also be expressed by Golgi cells (white arrows, Fig. 1 b, e). *Mef2b* expression, on the other hand, is not detected in the cerebellar cortex (Fig. 1c). *Mef2c* expression is restricted to the Purkinje cell layer, and not detected in the molecular or internal granular layer (Fig. 1d). Comparison with *Gad67*, a gene expressed by cerebellar GABAergic inhibitory neurons [37], shows that unlike *Mef2a* or *Mef2d* which are expressed in all three layers of the cerebellar cortex, *Mef2c* expression corresponds to *Gad67* in the Purkinje cell layer, indicating that *Mef2c* is specifically expressed by Purkinje cells (Fig. 1b–f).

**Fig. 2:**
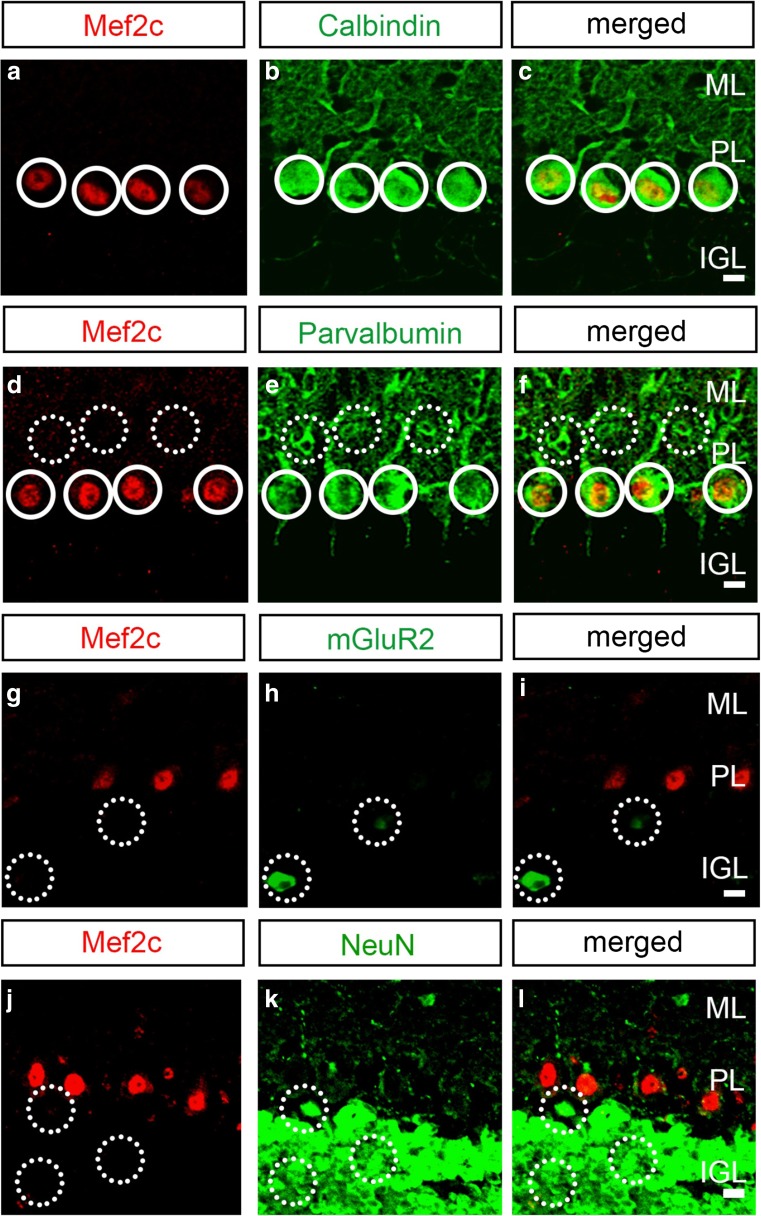
Mef2c is expressed specifically by Purkinje cells in the mature cerebellar cortex. **a**–**c** The expression of Mef2c (**a**, red) and calbindin (**b**, green) colocalizes within Purkinje cells (**c**, merge). **d**–**f** The expression of Mef2c (**d**, red) and parvalbumin (**e**, green) also colocalizes within Purkinje cells (**f**, merged), but not stellate/basket cells. **g**–**i** Mef2c expression (**g**, red) is absent in Golgi cells which are marked by mGluR2 (**h**, green) (**i**, merged). **j**–**l** The expression of Mef2c (**j**, red) also does not colocalize with NeuN (**k**, green) (**l**, merged) indicating a lack of expression in granule cells. Sagittal sections of lobule VIII from cerebellar vermis were used for this analysis. Complete circles represent cells with positive expression and broken circles indicate a lack of colocalization. PL, Purkinje cell layer; ML, molecular layer; IGL, internal granule layer. Age = P60. Scale bar = 10 μm

To confirm that the expression pattern of Mef2c protein is consistent with *Mef2c* RNA, we analyzed and compared the expression of Mef2c protein with known specific molecular markers of cerebellar neuronal subtypes. We first compared the expression of Mef2c with two calcium-binding proteins that define distinct GABAergic neuronal subpopulations in the cerebellar cortex: parvalbumin is expressed by stellate/basket cells in the molecular layer and Purkinje cells in the Purkinje cell layer, whereas calbindin is expressed only by Purkinje cells [38]. At P60, Mef2c colocalizes with calbindin and parvalbumin in the Purkinje cell layer, but not parvalbumin in the molecular layer (Fig. 2a–f). Comparison of the expression of Mef2c with zebrin, which defines Purkinje cells restricted to cerebellar zones [39], shows that the expression of Mef2c does not correspond with zebrin, indicating that Mef2c is expressed by most Purkinje cells (Fig. S1). Next, we assessed the colocalization pattern of Mef2c with mGluR2 and NeuN, which label Golgi and granule cells, respectively [40, 41], and found that the expression of Mef2c is not found in these two major neuronal subtypes in the internal granular layer, consistent with the lack of *Mef2c* RNA expression in granule cells and Golgi cells (Fig. 2g–l).

**Fig. 3:**
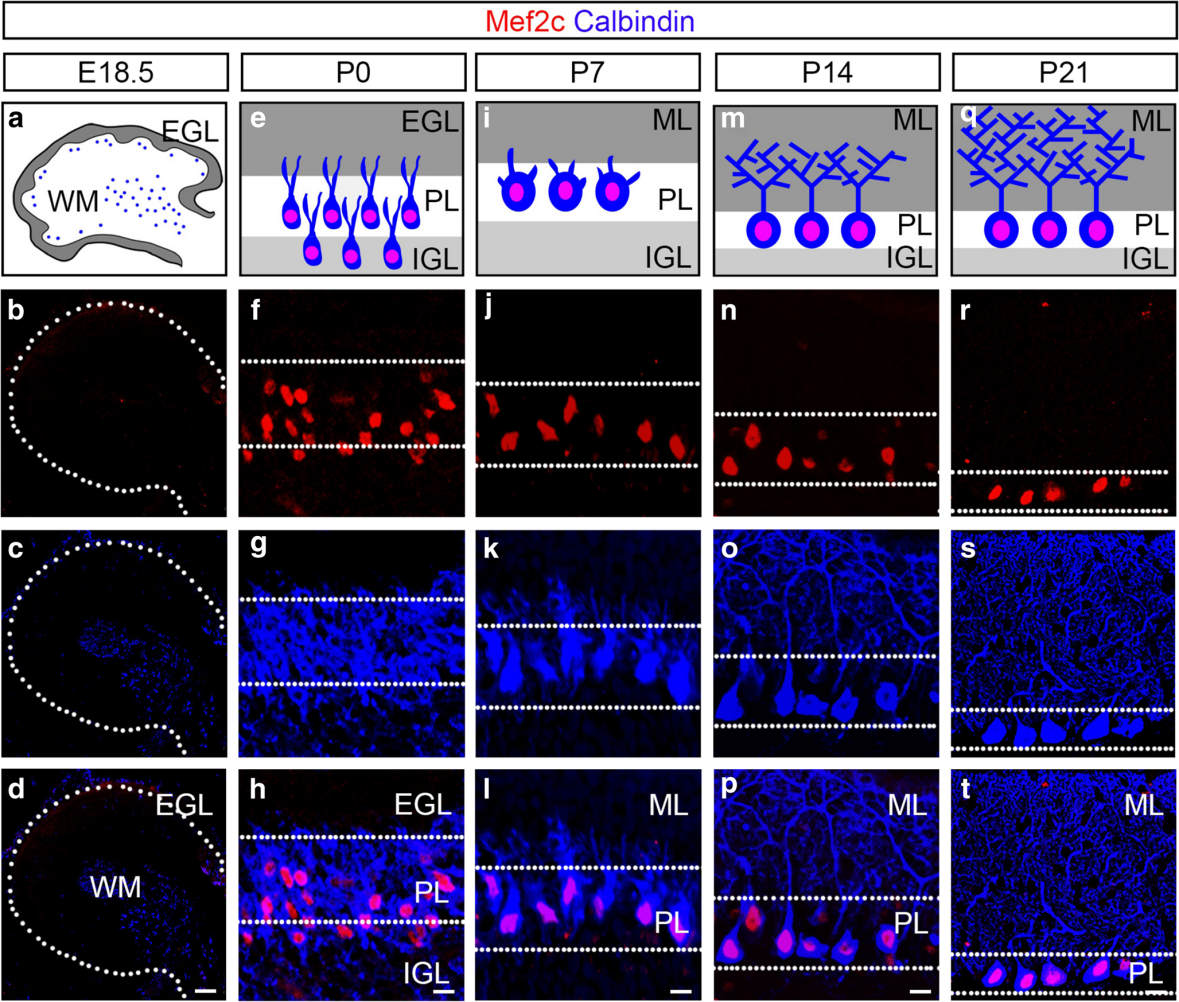
The onset of Mef2c expression in developing Purkinje cells. **a**–**d** The expression of calbindin (blue), but not Mef2c (red), is detected at E18.5. **e**–**h** Mef2c expression is first detected in Purkinje cells at P0. **i**–**t** Mef2c (red) continues to be expressed in calbindin^+^ Purkinje cells (blue) at P7 (**i**–**l**), P14 (**m**–**p**) and P21 (**q**–**t**). Merged (**d**, **h**, **l**, **p**, **t**). PL, Purkinje cell layer; ML, molecular layer; IGL, internal granule layer; E, embryonic day; P, postnatal day. Scale bar = 10 μm

To determine the developmental expression profile of Mef2c in Purkinje cells, we analyzed and compared the expression of Mef2c and calbindin at embryonic and key postnatal developmental stages of Purkinje cells [28]. After their initial generation at E10.5, Purkinje cells are found in the white matter layer where they migrate radially towards the cortical surface between embryonic day (E) 13-E17 [27, 42]. We observed that Mef2c is not detected at either E15.5 or E18.5, when calbindin^+^ Purkinje cells are localized in the white matter and early Purkinje cell layer (data not shown; Fig. 3a–d). During the first three postnatal weeks, Purkinje cells undergo local migration in the Purkinje cell layer to transit from multi-layer to a single layer and their dendrites undergo significant morphological changes [28]. At P0, we first detected colocalization of Mef2c with calbindin (Fig. 3e–h). From P7 to P21, the expression of Mef2c persists in Purkinje cells as they merge to form a single layer and extend their dendrites into the molecular layer (Fig. 3i–t). Altogether, through the analysis of the expression of Mef2 family of transcription factors, we show that Mef2c is unique in its absence of expression in neurons in the molecular and internal granular layers. Moreover, our results indicate that the expression of Mef2c tightly correlates with the early postnatal development of Purkinje cells, raising the possibility that Mef2c controls the migration and/or dendritic morphogenesis of Purkinje cells.

### Consequences of Mef2c Knockdown on the Development of Purkinje Cells

**Fig. 4:**
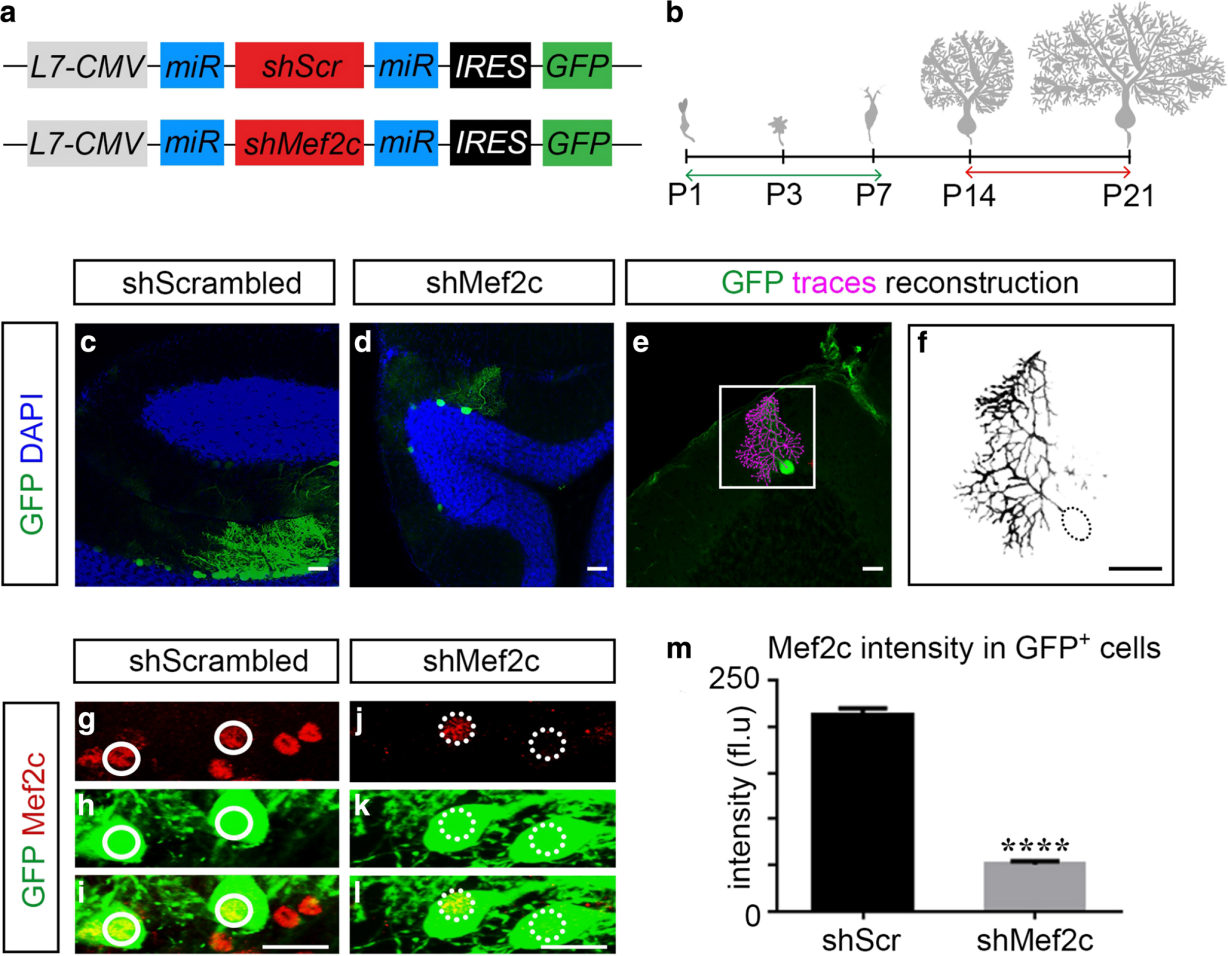
Selective labeling of Purkinje cells and shRNA-mediated knockdown of Mef2c. **a** Schematic diagram of the lentiviral shRNA constructs for control and Mef2c knockdown. **b** Schematic diagram showing key developmental stages of Purkinje cell dendrites. Green arrow indicates time of transduction (at P1, P3, and P7) and red arrow indicate time of analysis (at either P14 or P21). **c**–**d** Analysis of GFP (green) and DAPI (blue) in Purkinje cells after transduction with shScrambled (left) and shMef2c virus (right). **e**–**f** An infected Purkinje cells was traced (**e**, magenta) and reconstructed using the simple neurite tracer plugin in Fiji (**f**). **g**–**l** Analysis of Mef2c (red) in GFP^+^ Purkinje cells transduced with control (**g**–**i**) and knockdown lentivirus (**j**–**l)**. **m** shRNA-mediated knockdown of Mef2c results in reduction of the expression level of Mef2c (shScrambled 212.9 ± 6.913, *N* = 13, *n* = 38; shMef2c 50.48 ± 3.581, *N* = 15, *n* = 36, ****P < 0.0001). CMV, cytomegalovirus; miR, micro RNA; shRNA, short-hairpin RNA; IRES, internal ribosome entry site; GFP, green fluorescent protein; Data values = mean ± SEM, Student’s *t* test. Scale bar for **c**–**d** = 50 μm, **e**–**l** = 20 μm

Purkinje cells undergo dynamic morphological changes during the first three postnatal weeks [28, 43], and the coincidence of the onset and sustained expression of Mef2c within this period suggests that Mef2c may contribute to aspects of the development and maturation of Purkinje cells. In order to explore this possibility, we utilized lentiviral-mediated expression of shRNA to knockdown the expression of Mef2c in Purkinje cells. To restrict our genetic manipulation and analysis to only Purkinje cells, we used a minimal region of the L7 promoter to drive expression of a reporter and shRNA against Mef2c [32]. Short-hairpin RNA (shRNA) for control (scrambled) and for Mef2c knockdown were generated and placed downstream of the truncated L7-CMV promoter in a miRNA cassette (Fig. 4a). We infected Purkinje cells between P1–P7 and analyzed their morphological properties and organization within the cerebellar cortex at P14 or P21 (Fig. 4b). Infection of P1 pups with lentiviral constructs results in specific expression of GFP in Purkinje cells and permits the visualization and reconstruction of the dendritic pattern (Fig. 4c–f). We analyzed the level of Mef2c expression in GFP^+^ neurons (Fig. 4g–l) and found that shRNA-mediated Mef2c knockdown results in a ~three-fold decrease in Mef2c expression compared to control (Fig. 4m). The inability of Mef2c shRNA to knockdown Mef2a indicates that it is capable of selectively reducing the level of Mef2c (Fig. S4). Thus, we show that lentiviral-mediated transduction in combination with the use of the L7 minimal promoter permits both efficient knockdown of Mef2c and analysis of the functional relevance of Mef2c specifically in developing Purkinje cells.

The soma size of Purkinje cells increases rapidly and multiple dendrites are pruned into one single primary dendrite during the first two postnatal weeks of cerebellar development [28, 44]; and, the soma size and initial dendritic pruning of Purkinje cells have been shown to be dependent on transcription factors [23, 45]. We first examined whether Mef2c knockdown influences the general morphological properties of Purkinje cells and found that the mean soma size of Purkinje cell soma at P21 is not affected by Mef2c knockdown at P1 (Fig. S2a-f, h). A close examination of the primary dendrites of Purkinje cells also indicates that Mef2c knockdown does not result in improper pruning of perisomatic dendrites (Fig. S2a-f). Additionally, we did not find evidence of GFAP^+^ reactive gliosis, which has been associated with Purkinje cell death (Fig. S2i-l) [46]. The transition from multi-layers to a single layer of Purkinje cells occurs between P0 and P10 in the developing cerebellum [47]. Mef2c knockdown does not affect the formation of GFP^+^ Purkinje cells into a single layer or the position of GFP^+^ cells in relation to the edge of the molecular layer (Fig. S2m-t). Together, these results indicate that the loss of Mef2c does not influence the general growth or health of Purkinje cells and does not appear to play a role in the initial pruning of dendrites or postnatal migration of Purkinje cells.

#### Knockdown of Mef2c Expression Results in an Increase in Dendritic Complexity of Purkinje Cells

Studies of the underlying brain disruptions in neurological disorders, such as autism, in patients and mouse models have uncovered changes in the morphology of dendrites and the number of dendritic spines in Purkinje cells [22, 23, 48, 49]. The development and maintenance of dendritic morphology and spine density influence Purkinje cell function since extensive excitatory and inhibitory processes synapse on the elaborate dendritic tree [50]. After transitioning from multiple perisomatic processes to a single apical stem, rapid branching/elaboration of the apical stem dendritic tree takes place during the second and third postnatal week [28]. In order to determine whether and how Mef2c regulates the morphology of Purkinje cell dendrites, we knocked down Mef2 expression at two different stages and analyzed the dendritic tree of GFP^+^ Purkinje cells by Sholl analysis (see “Materials and Methods”).

**Fig. 5:**
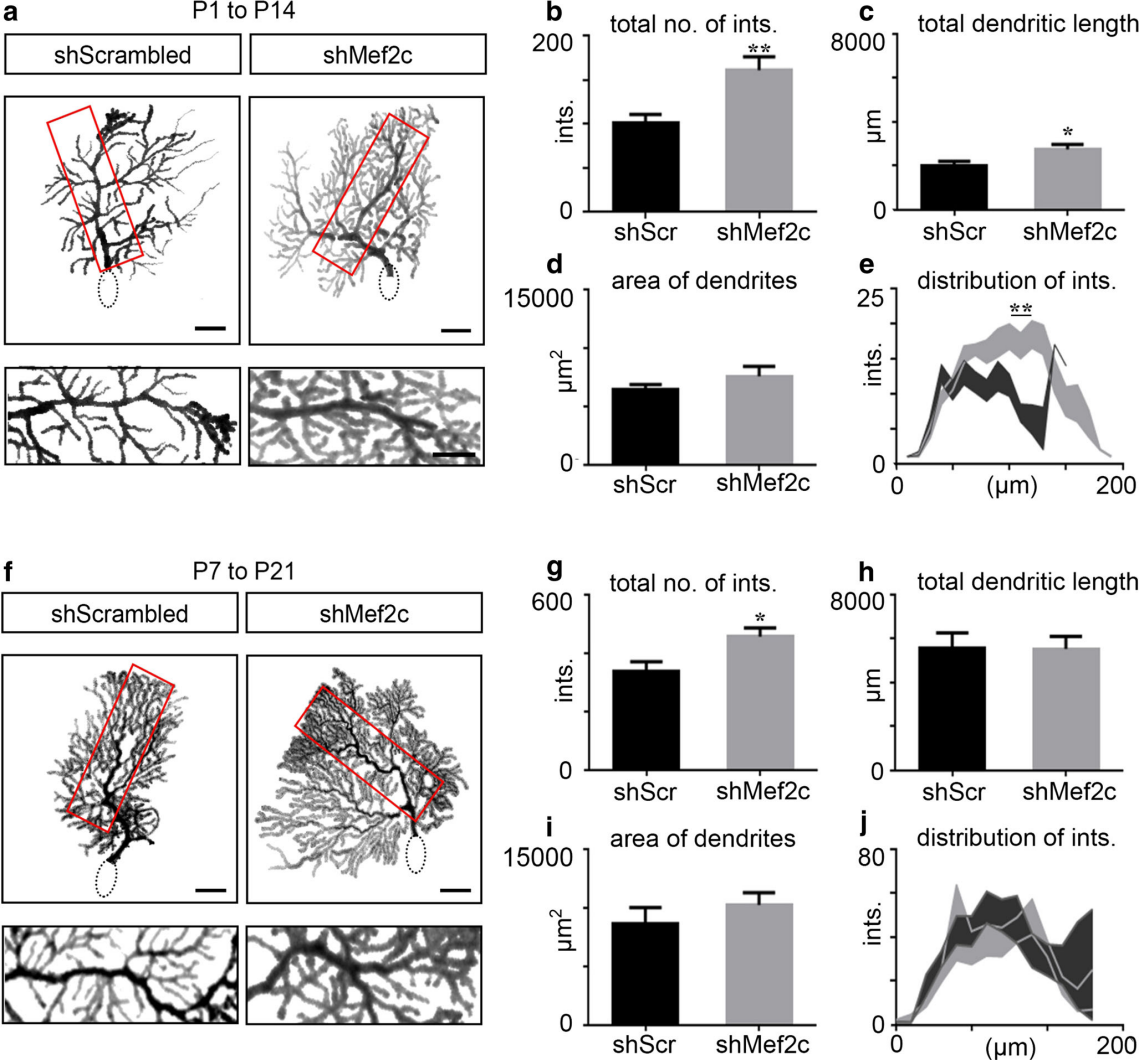
Mef2c knockdown results in an increase in complexity of Purkinje cell dendrites. **a** Representative traces of a control and shMef2c Purkinje cell at P14. **b** Analysis of the total number of intersections in control (black) and Mef2c knockdown (gray) Purkinje cells (shScrambled 100.5 ± 10.34, *n* = 11; shMef2c 161.3 ± 14.69, *n* = 18; **P = 0.0063; *N* = 7 for shScrambled, 10 for shMef2c). **c** Analysis of the total dendritic length in control and Mef2c knockdown Purkinje cells (shScrambled 1974 ± 209.6, *n* = 10; shMef2c 2737 ± 222.4, *n* = 16; *P = 0.0285; *N* = 7 for shScrambled, 10 for shMef2c). **d** Analysis of the area of Purkinje cell dendrites (shScrambled 6393 ± 473.1, *n* = 14; shMef2c 7552 ± 852.8, *n* = 19; *P* = 0.2901; *N* = 7 for shScrambled, 10 for shMef2c). **e** Analysis of the distribution of dendritic intersections along the somato-dendritic axis shows a higher number of intersections in Mef2c knockdown Purkinje cells at 110 μm and 120 μm from the soma (at 110 μm **P = 0.0009; at 120 μm **P = 0.0008). **f** Representative traces of a control and shMef2c Purkinje cell at P21. **g** Analysis of the total number of intersections in control (black) and Mef2c knockdown (gray) Purkinje cells (shScrambled 107.2 ± 10.73, *n* = 5; shMef2c 294.5 ± 51.00, *n* = 4; *P = 0.005; *N* = 3 for shScrambled, 3 for shMef2c). **h** Analysis of the total dendritic length in control and Mef2c knockdown Purkinje cells (shScrambled 5576 ± 673.3, *n* = 6; shMef2c 5513 ± 576.9, n = 6; *P* = 0.9448; *N* = 3 for shScrambled, 3 for shMef2c). **i** Analysis of the area of Purkinje cell dendrites (shScrambled 8625 ± 1390, *n* = 9; shMef2c 10222 ± 1058, *n* = 8; *P* = 0.3844; *N* = 3 brains for shScrambled, 3 brains for shMef2c). **j** Analysis of the distribution of dendritic intersections along the somato-dendritic axis did not reveal any significant differences between control and shMef2c Purkinje cells. Scr, scrambled; PC, Purkinje cell; sh, short-hairpin RNA; ints., intersections; P, postnatal. Data values = mean ± SEM, Student’s *t* test. Scale bar = 20 μm

Knockdown of Mef2c expression at P1 results in a 61% increase in the total number of intersections from the single apical stem dendrite of Purkinje cells when analyzed at P14 (Fig. 5a, b). The total length of dendrites is increased by 38% without any changes to the area or height of the dendritic tree after Mef2c knockdown (Fig. 5 c, d; Fig. S3). Additionally, the distance of maximum intersections move from a more distal position (Fig. 5e; Fig. S3). In order to assess the contribution of Mef2c in Purkinje cell development beyond P14, and determine whether the effects of Mef2c knockdown on the morphology of Purkinje cells persists after P14, we analyzed the number of intersections and total length of dendritic trees at P21. Similar to the observations at P14, we saw a marked increase in the total number of intersections and dendritic length of Purkinje cell dendrites at P21 after knockdown of Mef2c at P1 (data not shown), indicating this morphological phenotype persists beyond the first two postnatal weeks. Because we did not see abnormal pruning of the multi-perisomatic dendrites after Mef2c knockdown (Fig. S2), this result suggests Mef2 selectively regulates branching, but not pruning, of Purkinje cell dendrites.

At the end of the first postnatal week, Purkinje cells begin to have a single apical dendritic stem and undergo an increase in branching over the next 2 weeks that corresponds to key developmental events, such as completion of parallel/climbing fiber elimination and formation of functional GABAergic synapses by stellate and basket cells [28]. In order to assess whether Mef2c has an ability to influence branching of Purkinje cell dendrites after the first postnatal week, we analyzed the dendritic trees of Purkinje cells at P21 after Mef2c knockdown at P7. Knockdown of Mef2c knockdown at P7 results in a 36% increase in the number of intersections of Purkinje cell dendrites (Fig. 5f, g). However, the total length and area of dendrites, as well as the distribution of intersections, are not significantly different between experimental groups (Fig. 5h–j; Fig. S3). Taken together, we show that the loss of Mef2c leads to an increase in branching of Purkinje cell dendrites, suggesting that Mef2c controls dendritic complexity by restricting the branching and arborization of Purkinje cell dendrites. Additionally, because the changes corresponding to Mef2c knockdown at P7 is less severe than knockdown at P1, there may be a critical period of the influence of Mef2c on dendritic development.

#### Knockdown of Mef2c Expression Results in an Increase in the Number of Terminal Spines and Changes in Excitatory and Inhibitory Synaptic Protein Localization on Purkinje Cell Dendrites

**Fig. 6:**
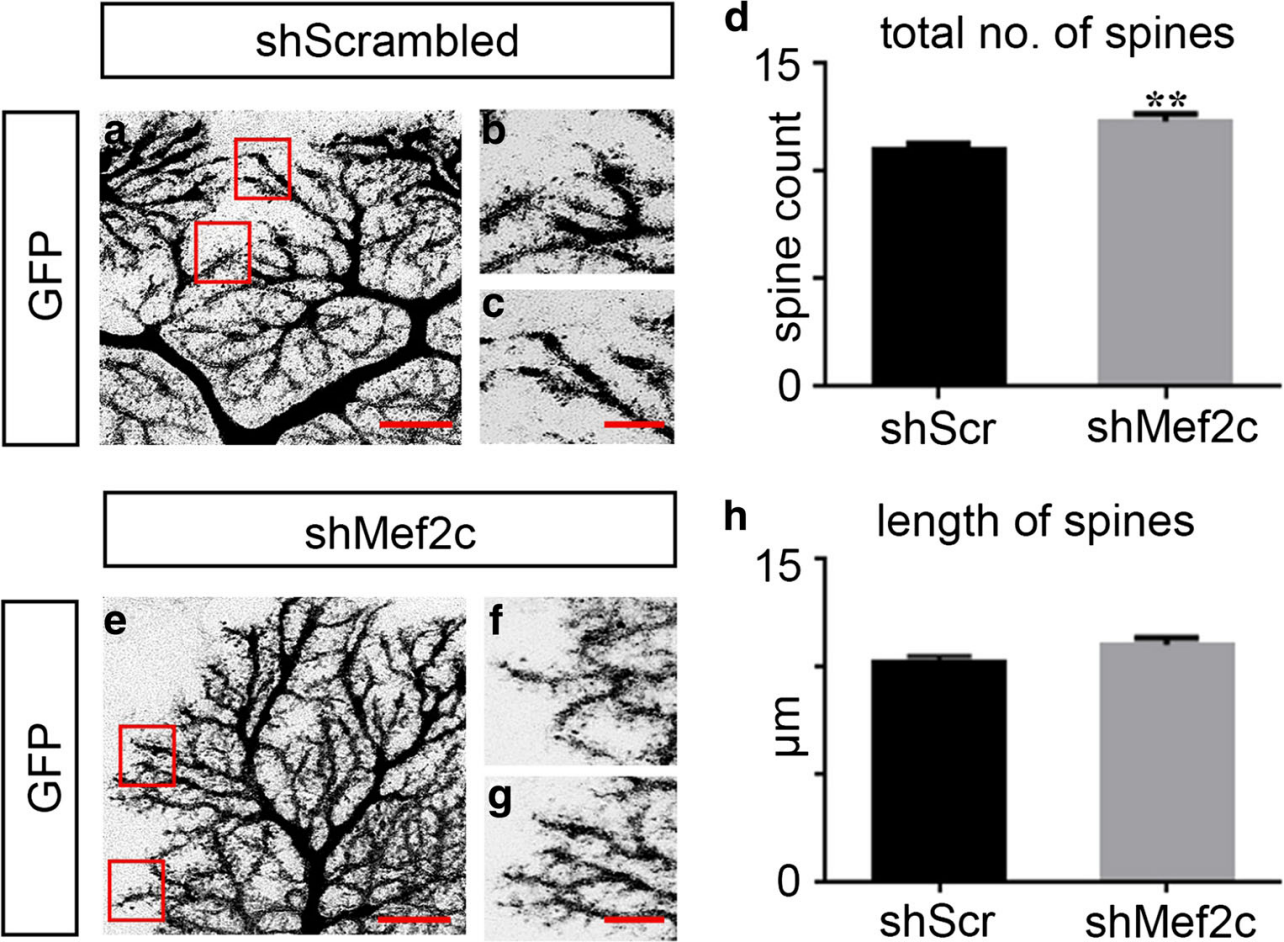
Mef2c knockdown results in an increase in number of spines on Purkinje cell dendrites. **a**–**c**, **e**–**g** Representative images of control (**a**, higher magnification in **b, c**) and shMef2c Purkinje cells (**e**, higher magnification in **f**, **g**). **d** Analysis of the total number of spines per 10-μm length of dendrite in control and shMef2c Purkinje cells (shScrambled 11.00 ± 0.2837, *n* = 30; shMef2c 12.33 ± 0.3368, *n* = 30; **P = 0.0037; *N* = 3 per experimental group). **h** Analysis of the average length of spines in control and shMef2c Purkinje cells (shScrambled 10.14 ± 0.2878, *n* = 60; shMef2c 10.94 ± 0.3413, *n* = 60; *P* = 0.0735; *N* = 3 per experimental group). Scr, scrambled, GFP, green fluorescent protein, sh, short-hairpin RNA. Data values = mean ± SEM, Student’s *t* test. Scale bar for **a**, **e** = 10 μm, **b**, **c**, **f**, **g** = 5 μm

Early postnatal spinogenesis of Purkinje cells can occur without excitatory input and is presumed to be regulated by cell-autonomous mechanisms [28]. Additionally, genetic manipulation of factors important for actin polymerization has shown that loss of spines can occur without any changes to the growth and morphology of the dendritic trees of Purkinje cells [28], suggesting spinogenesis and dendritic morphogenesis are regulated by independent pathways. To assess whether Mef2c plays a role in formation of spines, we knocked down Mef2c expression in P1 mice and assessed the number and length of spines on terminal dendrites of GFP^+^ neuron at P14. We were only able to detect spiney branchlets in GFP^+^ neurons even though Purkinje cells are known to possess two types of spines: thorns, sites of climbing fiber contacts located mostly on primary and secondary dendrites, and spiney branchlets, sites of parallel fiber contacts found on tertiary and terminal dendrites [28]. The loss of Mef2c during the first postnatal week of Purkinje cell development results in a ~ 12% increase in the total number of spiny branchlets in the distal part of Purkinje dendritic tree compared to control (Fig. 6a–c, e–g, d). However, the loss of Mef2c does not impact the length of spines (Fig. 6h). Although only a modest effect on the number of spines is observed, our result indicates that the expression of Mef2c is required for maintenance of an appropriate density of Purkinje cell spines during early postnatal development.

**Fig. 7:**
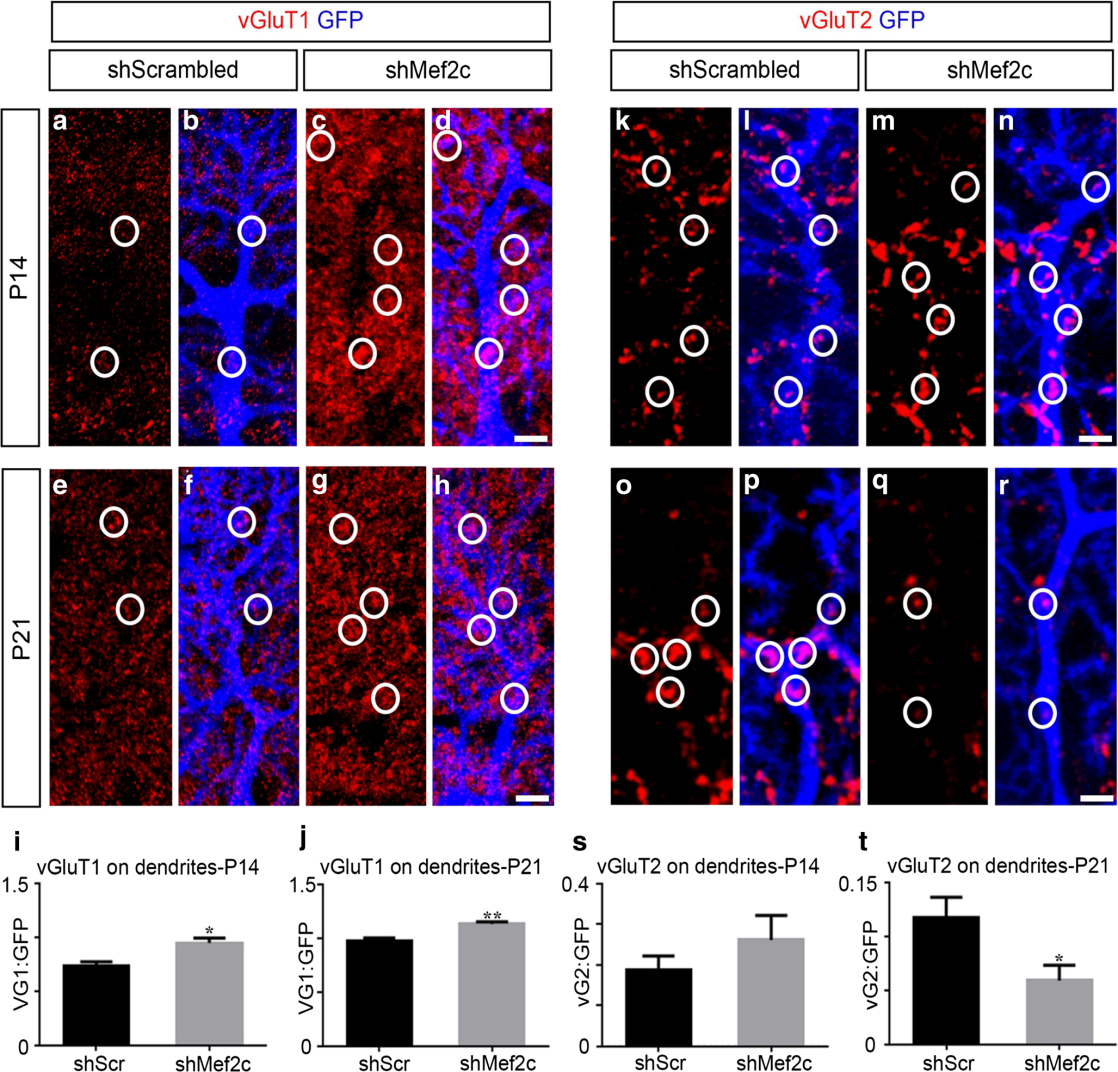
Mef2c knockdown results in an increase in vGluT1 and decrease in vGluT2 puncta on Purkinje cell dendrites. **a**–**d** Representative images of vGluT1 puncta (red, **a**) on GFP^+^ dendrites (blue, **b**) of a control Purkinje cell, and vGluT1 puncta (red, **c**) on GFP^+^ dendrites (blue, **d**) of a shMef2c Purkinje cell at P14 after viral transduction at P1. **i** Analysis of the coincidence of vGluT1 puncta on the dendrites of control and shMef2c Purkinje cells expressed as a ratio (shScrambled 0.7360 ± 0.03347, *n* = 10; shMef2c 0.9460 ± 0.04532, *n* = 10; *P = 0.0015; *N* = 4 for shScrambled; 5 for shMef2c). **e**–**h** Representative images of vGluT1 puncta (red, **e**) on GFP^+^ dendrites (blue, **f**) of a control Purkinje cell, and vGluT1 puncta (red, **g**) on GFP^+^ dendrites (blue, **h**) of a shMef2c Purkinje cell at P21 after viral transduction at P1. **i** Analysis of the coincidence of vGluT1 puncta on the dendrites of control and shMef2c Purkinje cells expressed as a ratio (shScrambled 0.9760 ± 0.02477, *n* = 18; shMef2c 1.129 ± 0.01975, *n* = 16; *P = 0.0001; *N* = 5 for shScrambled; 5 for shMef2c). **k**–**n** Representative images of vGluT2 puncta (red, **k**) on GFP^+^ dendrites (blue, **l**) of a control Purkinje cell, and vGluT2 puncta (red, **m**) on GFP^+^ dendrites (blue, **n**) of a shMef2c Purkinje cell at P14 after viral transduction at P1. **s** Analysis of the coincidence of vGluT2 puncta on the dendrites of control and shMef2c Purkinje cells expressed as a ratio (shScrambled 0.1880 ± 0.03289, *n* = 10; shMef2c 0.2607 ± 0.06061, *n* = 15; *P* = 0.3693; *N* = 5 for shScrambled; 7 for shMef2c). **o**–**r** Representative images of vGluT2 puncta (red, **o**) on GFP^+^ dendrites (blue, **p**) of a control Purkinje cell, and vGluT2 puncta (red, **q**) on GFP^+^ dendrites (blue, **r**) of a shMef2c Purkinje cell at P21 after viral transduction at P1. **t** Analysis of the coincidence of vGluT2 puncta on the dendrites of control and shMef2c Purkinje cells expressed as a ratio (shScrambled 0.1167 ± 0.01944, *n* = 12; shMef2c 0.05875 ± 0.01432, *n* = 15; *P = 0.0433; *N* = 5 for shScrambled; 7 for shMef2c). vGluT1/ vG1, vesicular glutamate transporter 1; vGluT2/ vG2, vesicular glutamate transporter 2. Data values = mean ± SEM, Student’s *t* test. Scale bar = 5 μm

Loss of Mef2c leads to an imbalance of excitatory and inhibitory synapse densities in cortical and hippocampal neurons [6, 34]; however, these studies focused their analysis only on excitatory neurons. In order to determine whether Mef2c plays a similar role in GABAergic neurons, we analyzed the localization of vGluT1 and vGluT2 on GFP^+^ Purkinje cell dendrites. vGluT1 and vGluT2 are molecular markers for glutamatergic parallel and climbing fiber synaptic puncta, respectively [51]. We observed that the loss of Mef2c resulted in a ~ 28% increase of vGluT1 puncta at P14 and a ~ 15% increase in vGluT1 puncta at P21 on GFP^+^ Purkinje cell dendrites (Fig. 7a–j). Mef2c knockdown at P1 did not result in significant changes of vGluT2 puncta localization at P14 (Fig. 7k–n, s), but lead to a ~ 50% reduction of vGluT2 puncta at P21 compared to control (Fig. 7o–r, t). The localization of both vGluT1 and vGluT2 puncta on Purkinje cell soma is not affected after Mef2c knockdown compared to control (Fig. S5 a-t). Thus, our results indicate a requirement of Mef2c in a GABAergic neuronal subtype in the maintenance of proper excitatory input to Purkinje cells.

**Fig. 8:**
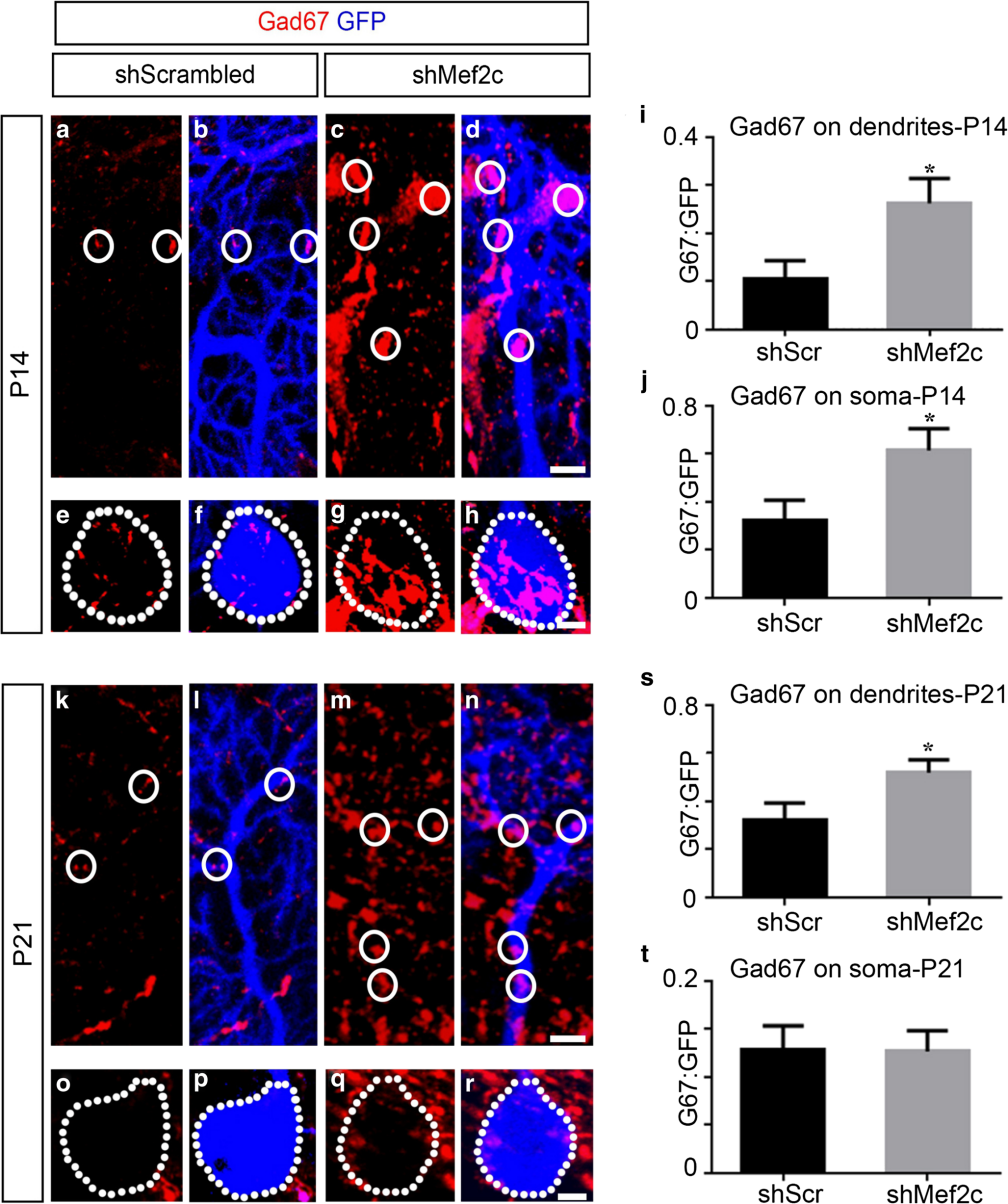
Loss of Mef2c results in an increase in Gad67 puncta to the soma and dendrites of Purkinje cells at P14 and only to the dendrites at P21. **a**–**h** Representative images of Gad67 puncta (red, **a, e**) on GFP^+^ dendrites (blue, **b**) and soma (blue, **e**) of a control Purkinje cell, and Gad67 puncta (red, **c**, **g**) on GFP^+^ dendrites (blue, **d**) and soma (blue, **h**) of a shMef2c Purkinje cell at P14 after viral transduction at P1. **i** Analysis of the coincidence of Gad67 puncta on the dendrites of control and shMef2c Purkinje cells expressed as a ratio (shScrambled 0.1044 ± 0.03779, *n* = 19; shMef2c 0.2619 ± 0.05124, *n* = 16; *P = 0.0453; *N* = 9 for shScrambled; 12 for shMef2c). **j** Analysis of the coincidence of Gad67 puncta on the soma of control and shMef2c Purkinje cells expressed as a ratio (shScrambled 0.3250 ± 0.08288, *n* = 18; 0.6150 ± 0.09095, *n* = 16; *P = 0.0390; *N* = 9 for shScrambled; 12 for shMef2c). **k**–**r** Representative images of Gad67 puncta (red, **k**, **o**) on GFP^+^ dendrites (blue, **l**) and soma (blue, **p**) of a control Purkinje cell, and Gad67 puncta (red, **m**, **q**) on GFP^+^ dendrites (blue, **n**) and soma (blue, **r**) of a shMef2c Purkinje cell at P21 after viral transduction at P1. **s** Analysis of the coincidence of Gad67 puncta on the dendrites of control and shMef2c Purkinje cells expressed as a ratio (shScrambled 0.3238 ± 0.07044, *n* = 22; shMef2c 0.5184 ± 0.05366, *n* = 25; *P = 0.0321; *N* = 11 for shScrambled; 10 for shMef2c). **t** Analysis of the coincidence of Gad67 puncta on the soma of control and shMef2c Purkinje cells expressed as a ratio (shScrambled 0.1283 ± 0.02519, *n* = 21; shMef2c 0.1267 ± 0.02175, *n* = 25; *P* = 0.9603; *N* = 11 for shScrambled; 10 for shMef2c). Gad67, glutamate decarboxylase 67. Data values = mean ± SEM, Student’s *t* test. Scale bar = 5 μm

In order to examine consequences of Mef2c knockdown at P1 on inhibitory inputs on Purkinje cells, we analyzed the incidence of Gad67 puncta on GFP^+^ Purkinje cells at P14 and P21. At P14, we observed a ~ 2.6-fold increase in Gad67 puncta on Purkinje cell dendrites (Fig. 8 a–d, i), and a 2-fold increase in Gad67 puncta after Mef2c knockdown compared to control (Fig. 8e–h, j). At P21, the loss of Mef2c did not result in significant changes in Gad67 puncta localization in the soma (Fig. 8o–r, t), but resulted in a ~ 66% increase in Gad67 puncta compared to scrambled (Fig. 8k–n, s). Thus, our results indicate that Mef2c has the ability to regulate both inhibitory and excitatory input onto Purkinje cells and may play an important role in maintaining the proper balance between parallel and climbing fiber input in developing and mature Purkinje cells. Taken together, we provide evidence that the specific expression of Mef2c is critical for maintaining the complexity of Purkinje cell dendrites during the first three postnatal weeks of development and is an important regulator of synaptic input.

## Discussion

In this study, we examined the role of Mef2c, a transcription factor implicated in a number of neurological disorders through characterization of the expression and functional relevance of Mef2c in the developing cerebellar cortex. We found that Mef2c is expressed specifically in Purkinje cells and that the onset of Mef2c expression occurs between E18.5 and P0 and persists into adulthood. The loss of Mef2c expression during the first or second postnatal week results in an increase in dendritic arborization, without impact on the general growth or migration of Purkinje cells. The loss of Mef2c expression resulting in an increase in the number of spines and changes in both excitatory and inhibitory puncta localization is suggestive of a potential imbalance in excitatory and/or inhibitory synaptic input. Thus, our study provides details of the specific expression and function of Mef2c in cerebellar Purkinje cells and lays the groundwork for future examination of the behavioral and electrophysiological consequences of the specific manipulation of Purkinje cell dendritic tree. Moreover, we demonstrate that the reduced expression of a transcription factor implicated in neurological disorders in a GABAergic neuronal subtype may perturb neuronal connectivity, which could lead to pathogenesis of cerebellar-associated disorders.

### Mef2c Is an Essential Regulator of the Development of Purkinje Cell Dendrites and Spines

Transcription factors play critical roles in the specification of neuronal subtypes and regulation of morphology and synaptic formation in the developing nervous system [52–54], and many continue to exert their influence in an activity-dependent manner for the maintenance and refinement of neuronal processes in mature animals [55–57]. In comparison to the large number of transcriptional programs important for generation and development of excitatory granule cells in the cerebellum [58–62], relatively few transcription factors have been identified for cerebellar inhibitory neurons. The specification and differentiation of cerebellar GABAergic neurons require transcription factors Ptf1a and Tfap2a/b [63–65]. The generation and differentiation of Purkinje cells depend on Olig2, Lhx1/5, Corl2/Skor2, and RORα; however, the expression of Olig2 is turned off during embryonic stages and the expression of Lhx1/5 and RORα is not restricted to Purkinje cells [45, 66–69]. Similar to Corl2/Skor2, our characterization of the expression and functional relevance of Mef2c provides a genetic entry point for the specific interrogation of Purkinje cell differentiation and Purkinje cell-mediated function. Moreover, our study provides evidence that in addition to excitatory neurons, Mef2c controls the development and, perhaps, maintenance of inhibitory neuronal processes.

The assembly of functional circuits in the brain requires proper dendritic formation and morphogenesis for integration of synaptic input, and these processes are tightly regulated by a number of transcription factors [70]. Even though Purkinje cells are born during embryonic stages, the growth and expansion of their dendritic tree occur during the first 3 weeks in postnatal mice [71]. We show that the onset of Mef2c expression coincides with the period of dynamic change in Purkinje cell dendrite development (Fig. 2) and provide evidence that the expression of Mef2c is critical for regulating the complexity of dendritic patterns. Because the expression of Corl2/Skor2 is turned on at E12.5, in addition to dendrite development, Corl2/Skor2 is required for early specification and migration of Purkinje cells [66, 67]. Similarly, RORα expression comes on in Purkinje cells at E13 and regulates the survival of Purkinje cells as well as the pruning, formation, and maintenance of dendrites [45, 72]. In contrast to both Corl2/Skor2 and RORα, the loss of Mef2c from Purkinje cells disrupts dendritic and spine formation without affecting migration, somatic pruning, or survival. Additionally, the loss of RORα results in a decrease in dendritic complexity, whereas the loss of Mef2c results in an increase in complexity, suggesting opposing roles of these two transcription factors. Therefore, likely due to postnatal onset of expression, Mef2c appears to be a specific regulator of dendritic formation and morphogenesis.

### Distinct Expression and Function of Mef2 Family of Transcription Factors in the Developing Cerebellum

The cerebellum has been identified as one of the brain regions of humans and mice with high expression level of Mef2 family of transcription factors [13, 14, 36, 73, 74], suggesting that they coordinate aspects of development and maintenance of the cerebellum. With the exception of Mef2b, all members of this family are expressed in the mouse cerebellum [36, 73, 74]. The expression of both Mef2a and Mef2d are first detected in the cerebellum at late embryonic stages in Purkinje and granule cells consistent with our results (Fig. S1) [73, 74], while Mef2c appears to be expressed predominantly by Purkinje cells [36]. However, the resolution of the analysis and lack of colocalization with neuronal subtype-specific molecular markers preclude conclusions about the specificity of Mef2c expression in Purkinje cells. For instance, whether Mef2 transcription factors are expressed in interneurons in the molecular and/or internal granular layer has not been determined. We observe that both Mef2a and Mef2d expression, but not Mef2c, are found in the molecular layer, and that Mef2a is expressed by Parvalbumin^+^ molecular layer interneurons (Fig. 1; data not shown). Importantly, our expression analysis provides a detailed description of the temporal and spatial expression of Mef2c in the cerebellum and reveals the postnatal onset and specific expression of Mef2c in Purkinje cells, which are distinct from Mef2a and Mef2d.

*Mef2* family of transcription factors carries out a multitude of functions ranging from neuronal proliferation, differentiation, and survival in various regions of the brain [4, 75]. In the cerebellum, studies have primarily focused on the function of Mef2 proteins in granule cells, but their role in Purkinje cells is largely unknown. Interference or knockdown of the expression of Mef2a and Mef2d results in reduced survival of cerebellar granule cells [76–80]. Additionally, the expression and transcriptional activity of Mef2a regulates dendritic differentiation of granule cells [8, 81] and localization of excitatory synaptic proteins onto granule cell dendrites [82]. In comparison to Mef2a and Mef2d, virtually nothing is known about the function of Mef2c in the cerebellum. The conventional deletion of Mef2a and Mef2c results in embryonic lethality due to cardiovascular defects, precluding analysis of brain development [83, 84]. Brain-specific deletion of Mef2a or Mef2a/d did not result in neuronal apoptosis, but the triple deletion of Mef2a/c/d did result in smaller brain size and increased cell death, suggesting redundancy in function of the Mef2 family members in regulation of neuronal survival [5, 11, 12]. Although mouse genetics studies link Mef2 with regulation of neuronal differentiation, survival, and synaptic transmission in the cerebral cortex and hippocampus [5, 11, 12], consequences of deletion of Mef2 genes on the development of the cerebellum have not yet been examined or reported. Thus, our identification of Mef2c in the regulation of dendrite development of Purkinje cells represents an important first step for a better understanding of the distinct roles of Mef2 transcription factors in the developing cerebellum.

Previous studies in defining the molecular mechanisms responsible for dendritic formation have identified a number of key regulators of calcium-mediated pathways including CDK5, CAMKII, and ERK [85]. All three of these regulators have been demonstrated to directly control the activity of Mef2 transcription factors [48], suggesting that one or more of these regulators could direct Mef2c-mediated dendritic morphogenesis of Purkinje cells. Additionally, Mef2c has been shown to exert its influence by employing downstream targets such as miRNAs and other translational modulators [86]. The identification and characterization of these molecules and factors with Purkinje cell-specific expression may provide mechanistic insight to how Mef2c controls morphological and synaptic organization of Purkinje cells. Our study provides evidence that Mef2c controls Purkinje cell-mediated functions by regulating the dendritic complexity and, perhaps, the number of excitatory synaptic inputs. Since disruption of the balance of excitation/inhibition in many regions of the brain has been linked to neurological disorders such as autism, which is associated with the disrupted number and efficacy of Purkinje cells [29], we propose that Mef2c serves as an important genetic entry point to better understand the contribution of Purkinje cells to these disorders.

### Mef2c Is a Critical Determinant of Excitatory and Inhibitory Connectivity in the Cerebellum

Climbing fiber synapses begin to form onto Purkinje cells at P3 and continue until P7 when they are selectively pruned until a single climbing fiber innervates one single Purkinje cell [28]. Although the exact time point of initiation has not been clearly defined, Purkinje cells also receive input from immature parallel fibers and possibly direct but transient mossy fiber input during the first postnatal week [51, 87]. Over the next 2 weeks, climbing fibers compete with parallel fibers to form defined innervation boundaries on each developing Purkinje cell [88]. From our analysis, we observe that by P21, Purkinje cells lacking Mef2c exhibit an increase in parallel fiber innervation and a decrease in climbing fiber innervation. This result raises the possibility that Mef2c may be involved in the process of climbing fiber pruning and/or competition between climbing and parallel fibers during development.

Previous studies have shown that the postnatal loss of Mef2c in hippocampal granule cells resulted in an increase in spine numbers [10], and the conditional deletion of Mef2c in the hippocampal and cortical excitatory neurons leads to a reduction of excitation and a simultaneous increase in inhibition [34]. These studies support a role of Mef2c in modulating the proper balance of excitation and inhibition through regulating spine properties. Purkinje cells receive two major excitatory inputs through parallel and climbing fiber connectivity [89]. During the early postnatal weeks of Purkinje cell development, parallel fibers compete with climbing fibers to define innervation boundaries within the cerebellar cortex and even within each individual Purkinje cell [88]. However, the molecular mechanisms underlying the process of defining climbing fiber-parallel fiber territories are not well understood. Our study revealed that the loss of Mef2c results in a reduction in climbing fiber innervation and a concurrent and, perhaps, compensatory increase in both parallel fiber and Gad67 input on dendrites of mature PCs. This is consistent with results by others that a reduction in climbing fiber input onto Purkinje cells resulted in a 2-fold and 4-fold increase in parallel fiber and Gad67 input, respectively [90]. Even though we see changes in the localization of synaptic puncta on Purkinje cells, there remains a possibility that Mef2c directly regulates the expression of vGluT1/2 and Gad67. Thus, Mef2c appears to be an important regulator of the assembly and maintenance of both excitatory and inhibitory inputs to PCs and a key determinant of proper circuit formation and function in the cerebellum.

## Electronic supplementary material

Fig. S1 **Mef2c expression does not correspond to zones defined by Zebrin. a.** The expression of Mef2c (red) in a coronal section of the cerebellum at P60. **b.** Schematic diagram of the corresponding cerebellar section. **c-h.** Comparison of the expression of Mef2c (red, **c, f**) and Zebrin (blue, **d, g**) (**e, h**, merged) in apical (**c-e**) and basal regions (**f-h**) of the cerebellar image from above. **i-k.** Comparison of the expression of Mef2c (red, **i**) and Calbindin (blue, **j**, merge in **k**) in the apical region of the cerebellar image from above. Abbreviations: SL, simple lobule; DCN, deep cerebellar nuclei; BA, basal; AP, apical; age = P60. Scale bar for **a** = 500 μm, **c**-**k** = 10 μm. (JPG 1332 kb)

Fig. S2 **Mef2c knockdown does not influence soma size, primary dendrite pruning or migration of Purkinje cells. a-f.** Representative images of GFP^+^ soma of control (**a-c**) and shMef2c Purkinje cells (**d-f**) at P14 after viral transduction at P3. **g.** Schematic diagram depicts the process of perisomatic dendrite pruning between P3-P14. **h.** Analysis of the soma size of control and shMef2c Purkinje cells (shScrambled: 290.7 ± 9.386, *n* = 20; shMef2c: 263.1 ± 18.49, n = 20; *P* = 0.1924). **i-l.** Representative images of the expression of GFAP (red) on or near GFP^+^ soma (green) of control (**i-j”**) and shMef2c Purkinje cells (**k-l”**) at P14 after viral transduction at P3. **m-r.** The organization and position of GFP^+^ (green) Calbindin^+^ (red) (blue, DAPI) control (**m-o**) and shMef2c Purkinje cells (**p-r**). **s.** Schematic diagram depicts the measurement of the distance between Purkinje cell soma and the outer edge of the molecular layer (Purkinje cells in black, and stellate/basket cells in gray). **t.** Analysis of the distance between the soma of GFP^+^ control and shMef2c Purkinje cells and the outer edge of the ML (shScrambled: 140.8 ± 3.080, *n* = 16; shMef2c: 140.5 ± 4.344, n = 16; *P* = 0.9555). Analysis was performed on Purkinje cells from lobules III to VIII, *N* = 12 for shScrambled and *N* = 16 for shMef2c. Abbreviations: GFAP, Glial fibrillary acidic protein. Scale bars: **a**-**f** = 5 μm, **i**-**l**’ = 5 μm, **m**-***r*** = 100 μm. (JPG 1790 kb)

Fig. S3 **Consequences of Mef2c knockdown on properties of Purkinje cell dendrites. a.** Analysis of the distance between the base of soma to maximum distance of dendrites in the molecular layer of control and shMef2c Purkinje cells at P14 after viral transduction at P1 (shScrambled: 108.2 ± 6.441, *n* = 11; shMef2c: 127.8 ± 7.434, *n* = 18; *P* = 0.0806; *N* = 7 for shScrambled, 10 for shMef2c). **b.** Analysis of the distance from the soma with highest number of intersections in control and shMef2c Purkinje cells at P14 after viral transduction at P1 (shScrambled: 100.5 ± 10.34, n = 11; shMef2c: 161.3 ± 14.69, n = 18; *P* = 0.0063; N = 7 for shScrambled, 10 for shMef2c). **c.** Analysis of the distance between the base of soma to maximum distance of dendrites in the molecular layer of control and shMef2c Purkinje cells at P21 after viral transduction at P7 (shScrambled: 117.5 ± 10.13, *n* = 8; shMef2c: 123.3 ± 14.98, *n* = 6; *P* = 0.7435). **d.** Analysis of the distance from the soma with highest number of intersections in control and shMef2c Purkinje cells at P21 after viral transduction at P7 (shScrambled: 57.50 ± 11.46, n = 8; shMef2c: 76.67 ± 16.87, n = 6; *P* = 0.3486.) (JPG 434 kb)

Fig. S4 **Mef2c shRNA does not reduce the level of Mef2a. a.** Western blot analysis for Mef2a and Gapdh using proteins samples obtained after transfection of overexpression and knockdown constructs in HEK293T cells. **b.** Graph showing the densitometry analysis of Mef2a, normalized with Gapdh (2a with 2a + sh2a: CI = 0.03702 to 0.1961, significant; 2a with 2a + sh2c: CI = −0.09654 to 0.06259, not significant). Abbreviations: 2a, overexpression of Mef2a; shSc, shRNA for scrambled; sh2a, shRNA for Mef2a; sh2c, shRNA for Mef2c; Ev, empty vectors; M2a, Mef2a; Gap, Gapdh; CI, confidence interval. Data values = mean ± SEM, One way Anova. (JPG 408 kb)

Fig. S5 **Loss of Mef2c does not affect vGluT1 and vGluT2 localization on Purkinje cell soma. a-h.** Representative images of vGluT1 puncta (red, **a**) on GFP^+^ soma (blue, **b**) of a control Purkinje cell, and vGluT1 puncta (red, **c**) on GFP^+^ soma (blue, **d**) of a shMef2c Purkinje cell at P14 after viral transduction at P1. **i.** Analysis of the coincidence of vGluT1 puncta on the soma of control and shMef2c Purkinje cells expressed as a ratio (shScrambled: 0.3670 ± 0.03055, shMef2c: *n* = 10; 0.3280 ± 0.02632, n = 10; *P* = 0.3463; *N* = 4 for shScrambled; 5 for shMef2c). **e-h.** Representative images of vGluT1 puncta (red, **e**) on GFP^+^ soma (blue, **f**) of a control Purkinje cell, and vGluT1 puncta (red, **g**) on GFP^+^ soma (blue, **h**) of a shMef2c Purkinje cell at P21 after viral transduction at P1. **j.** Analysis of the coincidence of vGluT1 puncta on the soma of control and shMef2c Purkinje cells expressed as a ratio (shScrambled: 0.4056 ± 0.02954, *n* = 18; shMef2c: 0.4144 ± 0.03473, *n* = 16; *P* = 0.8479; *N* = 5 for shScrambled; 5 for shMef2c) **k-r.** Representative images of vGluT2 puncta (red, **k**) on GFP^+^ soma (blue, **l**) of a control Purkinje cell, and vGluT2 puncta (red, **m**) on GFP^+^ soma (blue, **n**) of a shMef2c Purkinje cell at P14 after viral transduction at P1. **s.** Analysis of the coincidence of vGluT2 puncta on the soma of control and shMef2c Purkinje cells expressed as a ratio (shScrambled: 0.1850 ± 0.04458, n = 10; 0.1940 ± 0.02787, *n* = 15; *P* = 0.8581; N = 5 for shScrambled; 7 for shMef2c) **o-r.** Representative images of vGluT2 puncta (red, **o**) on GFP^+^ soma (blue, **p**) of a control Purkinje cell, and vGluT2 puncta (red, **q**) on GFP^+^ soma (blue, **r**) of a shMef2c Purkinje cell at P21 after viral transduction at P1. **t.** Analysis of the coincidence of vGluT2 puncta on the soma of control and shMef2c Purkinje cells expressed as a ratio (shScrambled: 0.1850 ± 0.04458, n = 10; shMef2c: 0.1940 ± 0.02787, n = 15; *P* = 0.8581; N = 5 for shScrambled; 7 for shMef2c). (shScrambled: 0.08833 ± 0.03200, *n* = 12; shMef2c: 0.0900 ± 0.02993, n = 10; *P* = 0.9705; *N* = 6 for shScrambled; 5 for shMef2c). Abbreviations: vGluT1/ vG1, vesicular glutamate transporter 1; vGluT2/ vG2, vesicular glutamate transporter 2. Data values = mean ± SEM, Student’s t test. Scale bar = 5 μm. (JPG 987 kb)
