# Supplementary figures and images for: Myocyte Enhancer Factor 2c Regulates Dendritic Complexity and Connectivity of Cerebellar Purkinje Cells
Source: Mol Neurobiol. 2018 Oct 1;56(6):4102–19. doi: 10.1007/s12035-018-1363-7 (PMC6505522; doi:10.1007/s12035-018-1363-7)

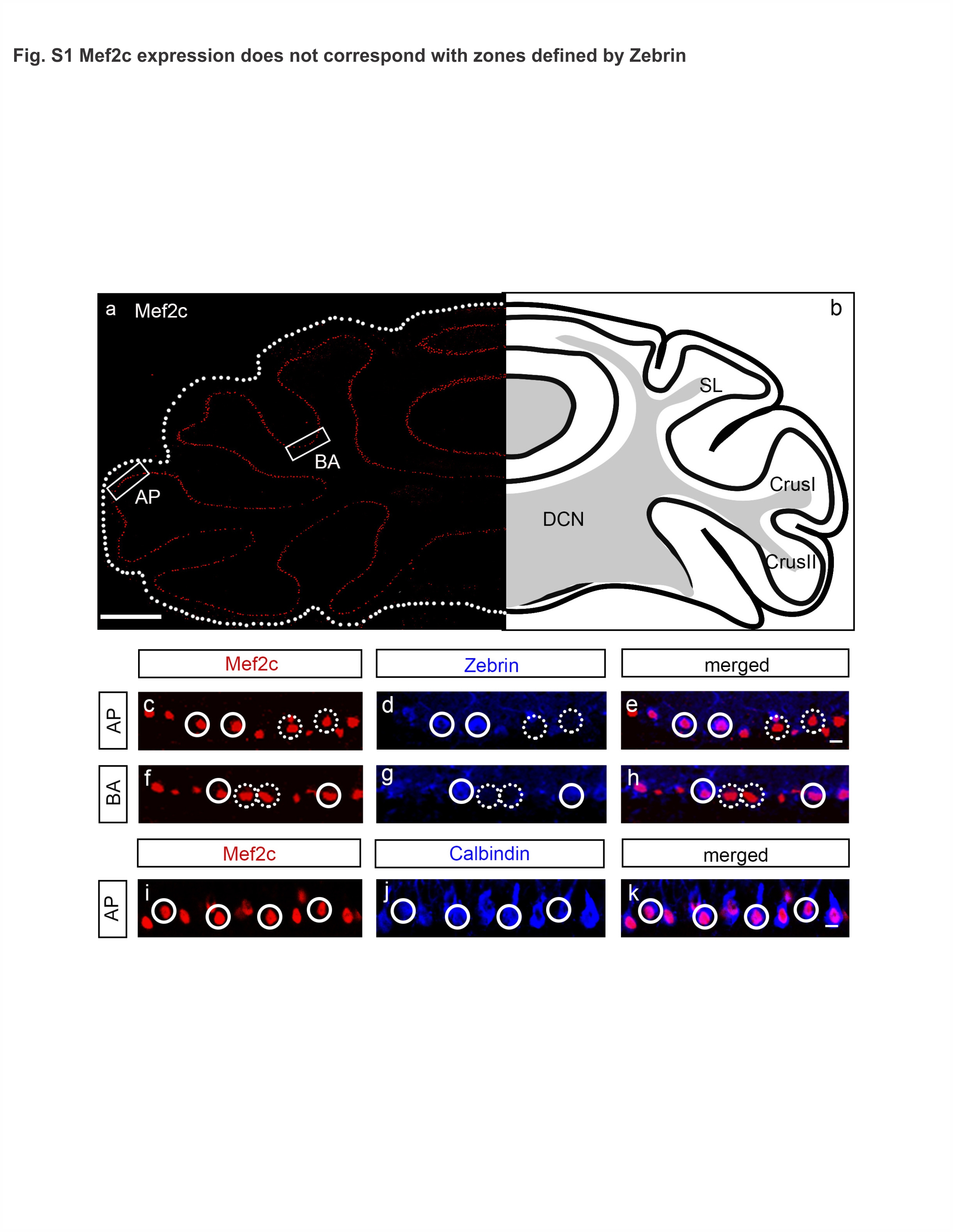

Supplement: Supplementary file 1 — Mef2c expression does not correspond to zones defined by Zebrin. a. The expression of Mef2c (red) in a coronal section of the cerebellum at P60. b. Schematic diagram of the corresponding cerebellar section. c-h. Comparison of the expression of Mef2c (red, c, f) and Zebrin (blue, d, g) (e, h, merged) in apical (c-e) and basal regions (f-h) of the cerebellar image from above. i-k. Comparison of the expression of Mef2c (red, i) and Calbindin (blue, j, merge in k) in the apical region of the cerebellar image from above. Abbreviations: SL, simple lobule; DCN, deep cerebellar nuclei; BA, basal; AP, apical; age = P60. Scale bar for a = 500 μm, c-k = 10 μm. (JPG 1332 kb) [file 12035_2018_1363_MOESM1_ESM.jpg]

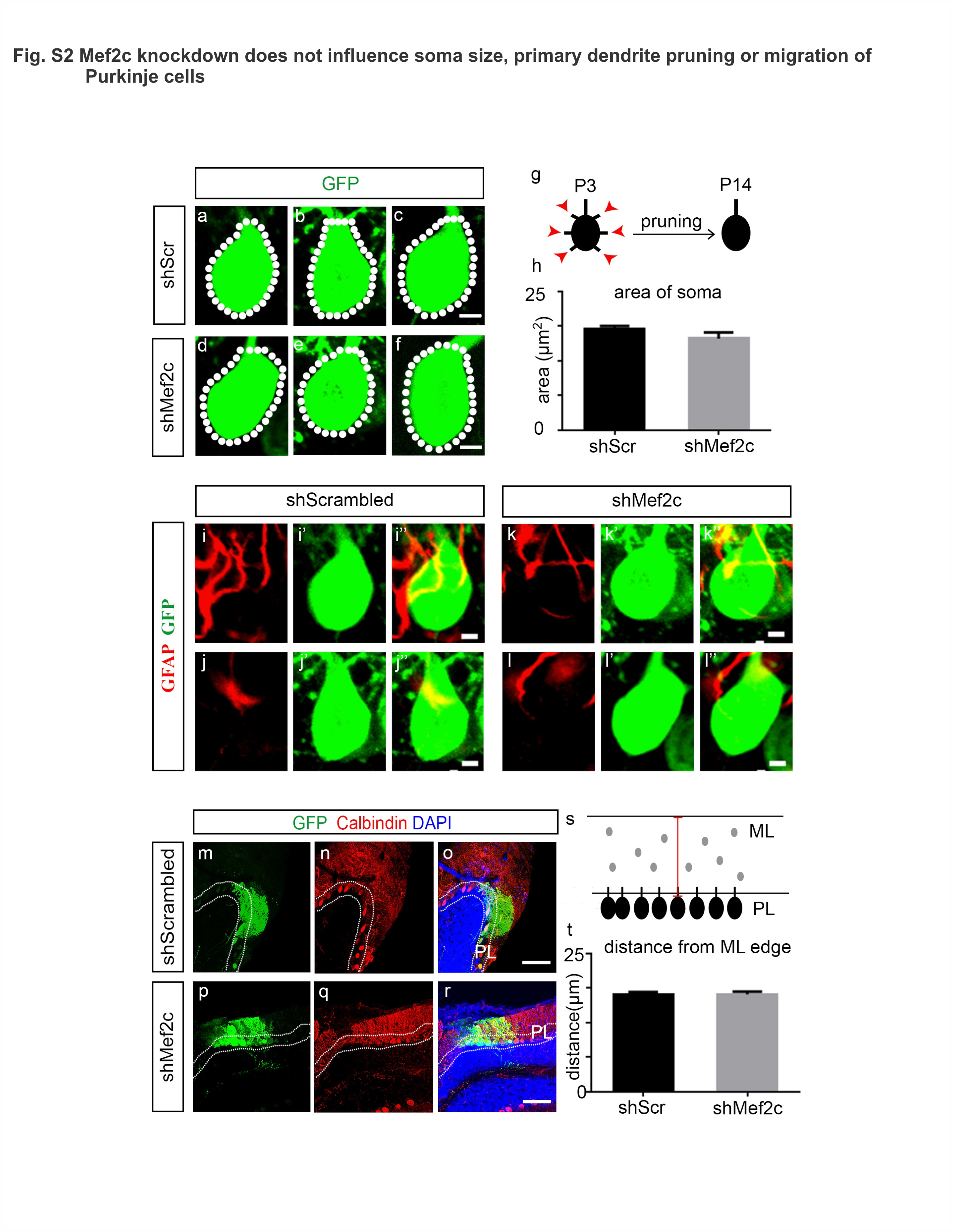

Supplement: Supplementary file 2 — Mef2c knockdown does not influence soma size, primary dendrite pruning or migration of Purkinje cells. a-f. Representative images of GFP+ soma of control (a-c) and shMef2c Purkinje cells (d-f) at P14 after viral transduction at P3. g. Schematic diagram depicts the process of perisomatic dendrite pruning between P3-P14. h. Analysis of the soma size of control and shMef2c Purkinje cells (shScrambled: 290.7 ± 9.386, n = 20; shMef2c: 263.1 ± 18.49, n = 20; P = 0.1924). i-l. Representative images of the expression of GFAP (red) on or near GFP+ soma (green) of control (i-j”) and shMef2c Purkinje cells (k-l”) at P14 after viral transduction at P3. m-r. The organization and position of GFP+ (green) Calbindin+ (red) (blue, DAPI) control (m-o) and shMef2c Purkinje cells (p-r). s. Schematic diagram depicts the measurement of the distance between Purkinje cell soma and the outer edge of the molecular layer (Purkinje cells in black, and stellate/basket cells in gray). t. Analysis of the distance between the soma of GFP+ control and shMef2c Purkinje cells and the outer edge of the ML (shScrambled: 140.8 ± 3.080, n = 16; shMef2c: 140.5 ± 4.344, n = 16; P = 0.9555). Analysis was performed on Purkinje cells from lobules III to VIII, N = 12 for shScrambled and N = 16 for shMef2c. Abbreviations: GFAP, Glial fibrillary acidic protein. Scale bars: a-f = 5 μm, i-l’ = 5 μm, m-r = 100 μm. (JPG 1790 kb) [file 12035_2018_1363_MOESM2_ESM.jpg]

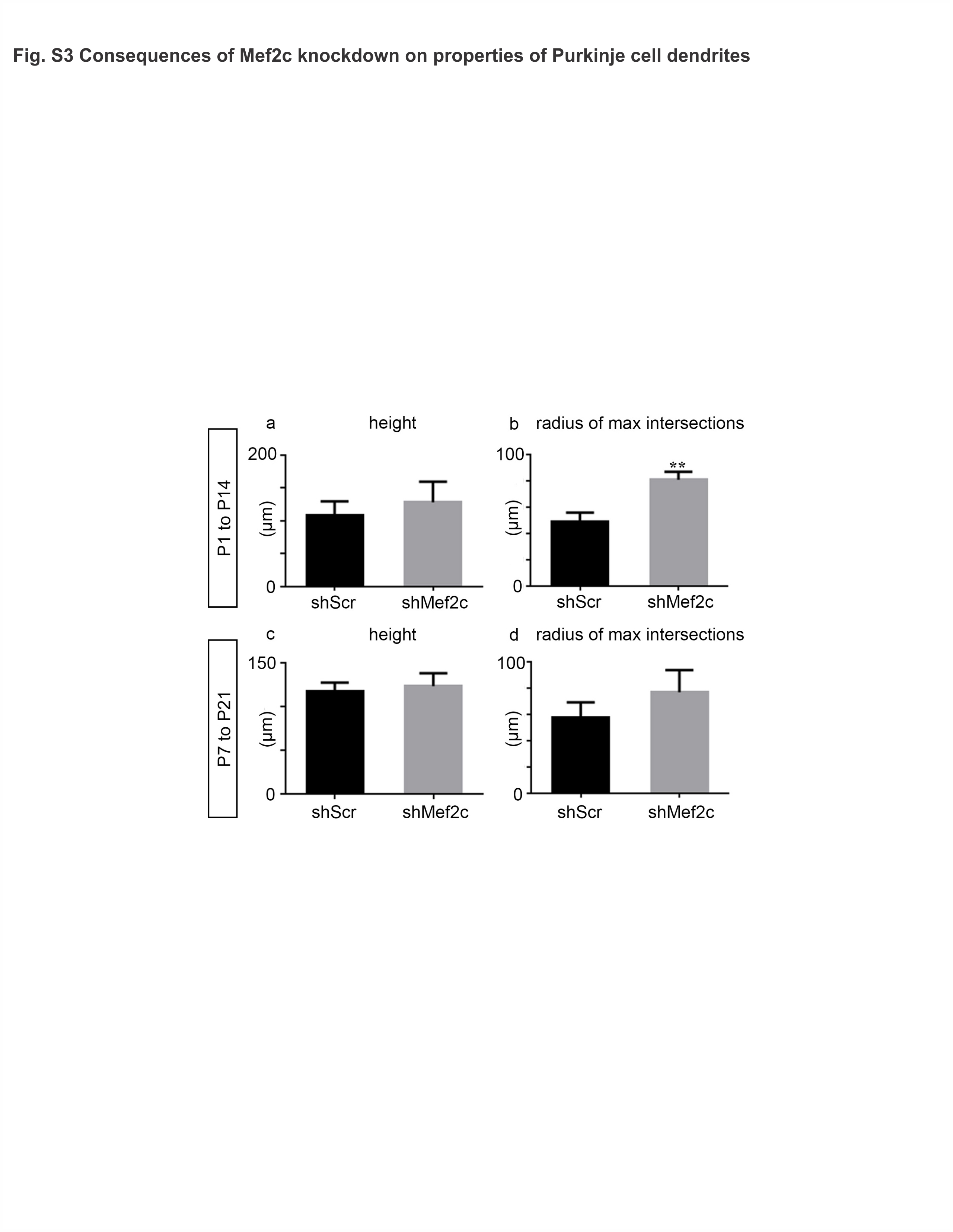

Supplement: Supplementary file 3 — Consequences of Mef2c knockdown on properties of Purkinje cell dendrites. a. Analysis of the distance between the base of soma to maximum distance of dendrites in the molecular layer of control and shMef2c Purkinje cells at P14 after viral transduction at P1 (shScrambled: 108.2 ± 6.441, n = 11; shMef2c: 127.8 ± 7.434, n = 18; P = 0.0806; N = 7 for shScrambled, 10 for shMef2c). b. Analysis of the distance from the soma with highest number of intersections in control and shMef2c Purkinje cells at P14 after viral transduction at P1 (shScrambled: 100.5 ± 10.34, n = 11; shMef2c: 161.3 ± 14.69, n = 18; P = 0.0063; N = 7 for shScrambled, 10 for shMef2c). c. Analysis of the distance between the base of soma to maximum distance of dendrites in the molecular layer of control and shMef2c Purkinje cells at P21 after viral transduction at P7 (shScrambled: 117.5 ± 10.13, n = 8; shMef2c: 123.3 ± 14.98, n = 6; P = 0.7435). d. Analysis of the distance from the soma with highest number of intersections in control and shMef2c Purkinje cells at P21 after viral transduction at P7 (shScrambled: 57.50 ± 11.46, n = 8; shMef2c: 76.67 ± 16.87, n = 6; P = 0.3486.) (JPG 434 kb) [file 12035_2018_1363_MOESM3_ESM.jpg]

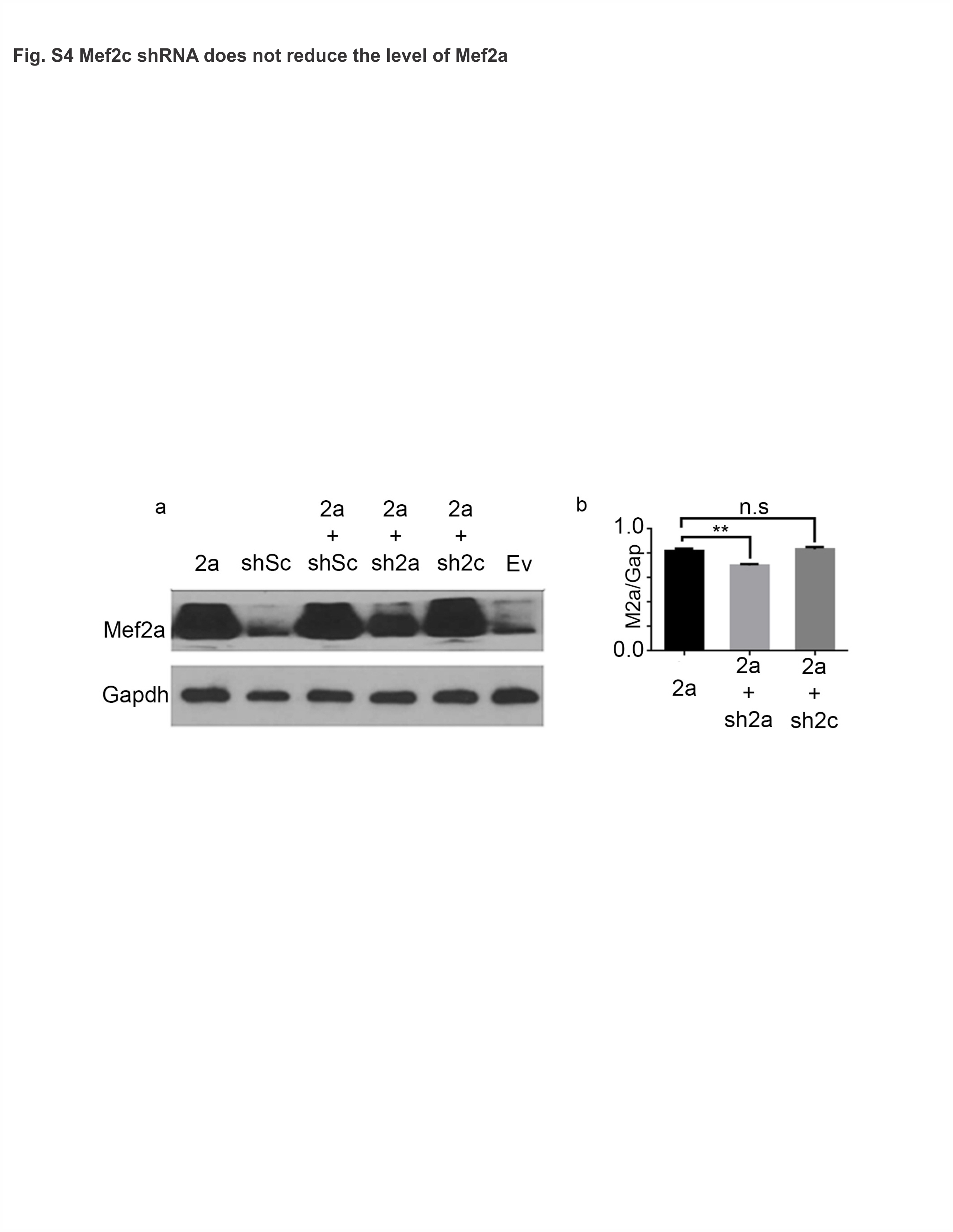

Supplement: Supplementary file 4 — Mef2c shRNA does not reduce the level of Mef2a. a. Western blot analysis for Mef2a and Gapdh using proteins samples obtained after transfection of overexpression and knockdown constructs in HEK293T cells. b. Graph showing the densitometry analysis of Mef2a, normalized with Gapdh (2a with 2a + sh2a: CI = 0.03702 to 0.1961, significant; 2a with 2a + sh2c: CI = −0.09654 to 0.06259, not significant). Abbreviations: 2a, overexpression of Mef2a; shSc, shRNA for scrambled; sh2a, shRNA for Mef2a; sh2c, shRNA for Mef2c; Ev, empty vectors; M2a, Mef2a; Gap, Gapdh; CI, confidence interval. Data values = mean ± SEM, One way Anova. (JPG 408 kb) [file 12035_2018_1363_MOESM4_ESM.jpg]

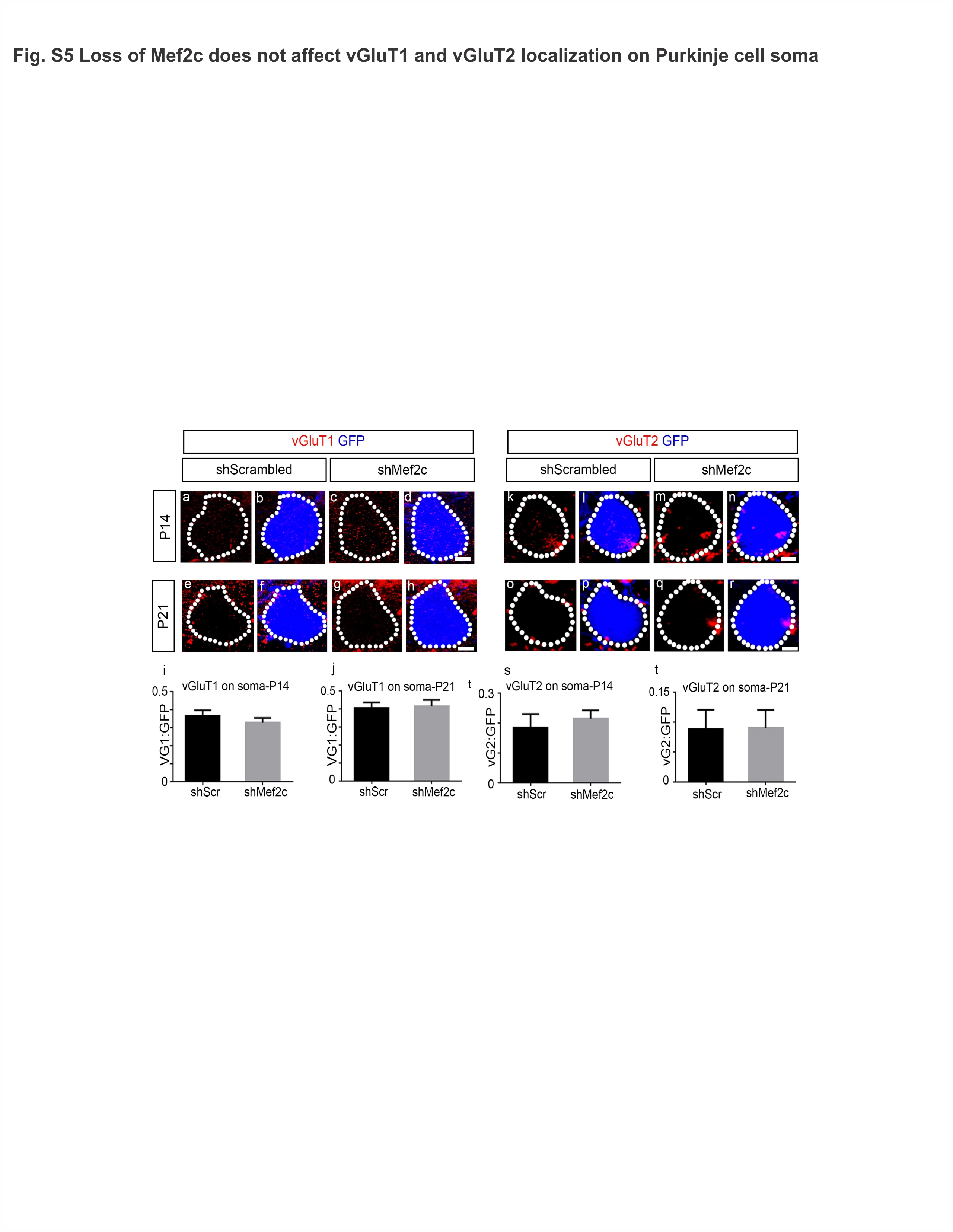

Supplement: Supplementary file 5 — Loss of Mef2c does not affect vGluT1 and vGluT2 localization on Purkinje cell soma. a-h. Representative images of vGluT1 puncta (red, a) on GFP+ soma (blue, b) of a control Purkinje cell, and vGluT1 puncta (red, c) on GFP+ soma (blue, d) of a shMef2c Purkinje cell at P14 after viral transduction at P1. i. Analysis of the coincidence of vGluT1 puncta on the soma of control and shMef2c Purkinje cells expressed as a ratio (shScrambled: 0.3670 ± 0.03055, shMef2c: n = 10; 0.3280 ± 0.02632, n = 10; P = 0.3463; N = 4 for shScrambled; 5 for shMef2c). e-h. Representative images of vGluT1 puncta (red, e) on GFP+ soma (blue, f) of a control Purkinje cell, and vGluT1 puncta (red, g) on GFP+ soma (blue, h) of a shMef2c Purkinje cell at P21 after viral transduction at P1. j. Analysis of the coincidence of vGluT1 puncta on the soma of control and shMef2c Purkinje cells expressed as a ratio (shScrambled: 0.4056 ± 0.02954, n = 18; shMef2c: 0.4144 ± 0.03473, n = 16; P = 0.8479; N = 5 for shScrambled; 5 for shMef2c) k-r. Representative images of vGluT2 puncta (red, k) on GFP+ soma (blue, l) of a control Purkinje cell, and vGluT2 puncta (red, m) on GFP+ soma (blue, n) of a shMef2c Purkinje cell at P14 after viral transduction at P1. s. Analysis of the coincidence of vGluT2 puncta on the soma of control and shMef2c Purkinje cells expressed as a ratio (shScrambled: 0.1850 ± 0.04458, n = 10; 0.1940 ± 0.02787, n = 15; P = 0.8581; N = 5 for shScrambled; 7 for shMef2c) o-r. Representative images of vGluT2 puncta (red, o) on GFP+ soma (blue, p) of a control Purkinje cell, and vGluT2 puncta (red, q) on GFP+ soma (blue, r) of a shMef2c Purkinje cell at P21 after viral transduction at P1. t. Analysis of the coincidence of vGluT2 puncta on the soma of control and shMef2c Purkinje cells expressed as a ratio (shScrambled: 0.1850 ± 0.04458, n = 10; shMef2c: 0.1940 ± 0.02787, n = 15; P = 0.8581; N = 5 for shScrambled; 7 for shMef2c). (shScrambled: 0.08833 ± 0.03200, n = 12; shMef2c: 0.0900 ± 0.02993, [file 12035_2018_1363_MOESM5_ESM.jpg]
